# Urbanization and genetic homogenization in the medieval Low Countries revealed through a ten-century paleogenomic study of the city of Sint-Truiden

**DOI:** 10.1186/s13059-025-03580-z

**Published:** 2025-05-20

**Authors:** Owyn Beneker, Ludovica Molinaro, Meriam Guellil, Stefania Sasso, Helja Kabral, Biancamaria Bonucci, Noah Gaens, Eugenia D’Atanasio, Massimo Mezzavilla, Hélios Delbrassine, Linde Braet, Bart Lambert, Pieterjan Deckers, Simone Andrea Biagini, Ruoyun Hui, Sara Becelaere, Jan Geypen, Maxim Hoebreckx, Birgit Berk, Petra Driesen, April Pijpelink, Philip van Damme, Sofie Vanhoutte, Natasja De Winter, Lehti Saag, Luca Pagani, Kristiina Tambets, Christiana L. Scheib, Maarten H. D. Larmuseau, Toomas Kivisild

**Affiliations:** 1 Department of Human Genetics, KU Leuven, Leuven, Belgium; 2 Department for Evolutionary Anthropology, University of Vienna, Vienna, Austria; 3 Human Evolution and Archaeological Sciences (HEAS), University of Vienna, Vienna, Austria; 4 Institute of Genomics, University of Tartu, Tartu, Estonia; 5 Institute of Molecular Biology and Pathology, CNR, Rome, Italy; 6 Department of Biology, University of Padova, Padova, Italy; 7 SHOC Research Group, Vrije Universiteit Brussel, Brussels, Belgium; 8 Archaeology, KU Leuven, Leuven, Belgium; 9 Department of Archaeology and Museology, Masaryk University, Brno, Czech Republic; 10 Center of Molecular Medicine, Central European Institute of Technology, Masaryk University, Brno, Czech Republic; 11 Alan Turing Institute, London, UK; 12 Histories vzw, Brussels, Belgium; 13 Aron bv, Bilzen, Belgium; 14 Birgit Berk Fysische Anthropologie, Meerssen, Netherlands; 15 Crematie en Inhumatie Analyse (CRINA) Fysische Antropologie, ’s-Hertogenbosch, Netherlands; 16 Department of Neurology, University Hospitals Leuven and Department of Neuroscience, KU Leuven, Leuven, Belgium; 17 Flanders Heritage Agency, Brussels, Belgium; 18 St John’s College, University of Cambridge, Cambridge, UK

**Keywords:** Urbanization, Palaeo-genomics, Low countries, Medieval, Migration, Plague, Flanders

## Abstract

**Background:**

Processes shaping the formation of the present-day population structure in highly urbanized Northern Europe are still poorly understood. Gaps remain in our understanding of when and how currently observable regional differences emerged and what impact city growth, migration, and disease pandemics during and after the Middle Ages had on these processes.

**Results:**

We perform low-coverage sequencing of the genomes of 338 individuals spanning the eighth to the eighteenth centuries in the city of Sint-Truiden in Flanders, in the northern part of Belgium. The early/high medieval Sint-Truiden population was more heterogeneous, having received migrants from Scotland or Ireland, and displayed less genetic relatedness than observed today between individuals in present-day Flanders. We find differences in gene variants associated with high vitamin D blood levels between individuals with Gaulish or Germanic ancestry. Although we find evidence of a *Yersinia pestis* infection in 5 of the 58 late medieval burials, we were unable to detect a major population-scale impact of the second plague pandemic on genetic diversity or on the elevated differentiation of immunity genes.

**Conclusions:**

This study reveals that the genetic homogenization process in a medieval city population in the Low Countries was protracted for centuries. Over time, the Sint-Truiden population became more similar to the current population of the surrounding Limburg province, likely as a result of reduced long-distance migration after the high medieval period, and the continuous process of local admixture of Germanic and Gaulish ancestries which formed the genetic cline observable today in the Low Countries.

**Supplementary Information:**

The online version contains supplementary material available at 10.1186/s13059-025-03580-z.

## Background

Compared to other world regions, Europe is genetically highly homogenous today, yet it displays fine-scale geographically correlated variation [1, 2] that can, if uncorrected, confound complex trait analyses [3]. The current interdisciplinary synthesis of evidence derived from archeology and genome-scale studies of ancient human remains explains allele frequency similarities across Europe by multiple prehistoric episodes of continent-wide population movements and admixture in the Neolithic and Bronze Age resulting in a more stable population structure in most parts of Europe since the Iron Age [4]. While the medieval period witnessed less gene flow to Europe from outside sources than previous periods, it was a time of major political changes and migration within the continental boundaries. The North Sea region, for example, witnessed major population movements in the Early Middle Ages (EMA) that led to massive changes in local genetic ancestries [5, 6]. In the same millennium, population movements in the Baltic Sea region contributed to the formation of regional population structure seen in the region today [7, 8]. On the other hand, evidence from Britain shows that local population structure continued to undergo extensive changes in the later medieval and post-medieval periods [9, 10]. The impact of demographic changes in continental western Europe during the last two millennia is less known due to the lack of relevant transect of time studies.

The Low Countries, consisting of Belgium, the Netherlands, and Luxembourg, have been since the medieval period one of the most densely populated and highly urbanized regions in Europe. Despite the homogenizing effects of population growth and migration in the last centuries, genetic differences between subregional populations are presently still clearly pronounced in the Low Countries, with an underlying north to south genetic ancestry cline that is likely to derive from admixture events dating at least to the eleventh century AD [11, 12]. In the absence of other obvious geographical boundaries, these regional patterns may have been shaped by the major rivers. Without direct genetic evidence with ancient DNA samples from a time transect through the Middle Ages, however, it remains unknown when and which historical events and ancestry sources have contributed to the formation of present-day population structure in the Low Countries.

The medieval history of the Low Countries, particularly in the now densely populated Flanders region of northern Belgium, is marked by extensive socio-political and economic developments that may have shaped the genetic landscape observed today. In this context, the town of Sint-Truiden, located in the modern province of Limburg, provides a particularly valuable case study as its history is well documented. The town’s origin is situated along two major Roman roads [13]. In the seventh century, Saint Trudo founded an abbey at the settlement called Sarchinium, which, after his death in 693, became a highly popular pilgrimage destination [14]. The influx of pilgrims played a crucial role in the growth of the settlement around the Benedictine abbey. By the twelfth c., this settlement had evolved into a thriving town named after its founder, Sint-Truiden. The town experienced strong expansion and population growth in the thirteenth c., largely due to its strategic location in the fertile agricultural region of Haspengouw and its specialization in the production of cloth and beer, which were traded internationally [13]. While historical scholarship has now acknowledged that *Yersinia pestis* circulated in the Low Countries from the Black Death (1346–1352) onwards, the chronicles of the abbey, *Gesta abbatum Trudonensium*, and the city’s administrative records do not mention any impact of the plague in the fourteenth c. [15, 16]. In the fifteenth c., historical evidence does refer to the town suffering from excess mortality and other problems likely caused by the plague [17]. The town was subject to intense political and military upheaval since the fourteenth c. and its decline was further exacerbated by the conquest of the town in 1467 during the conflict between the Burgundian State and the Prince-Bishopric of Liège [13]. Since then, Sint-Truiden has served as a regional center with a current population size of approximately 24,000 individuals within the city.

**Fig. 1:**
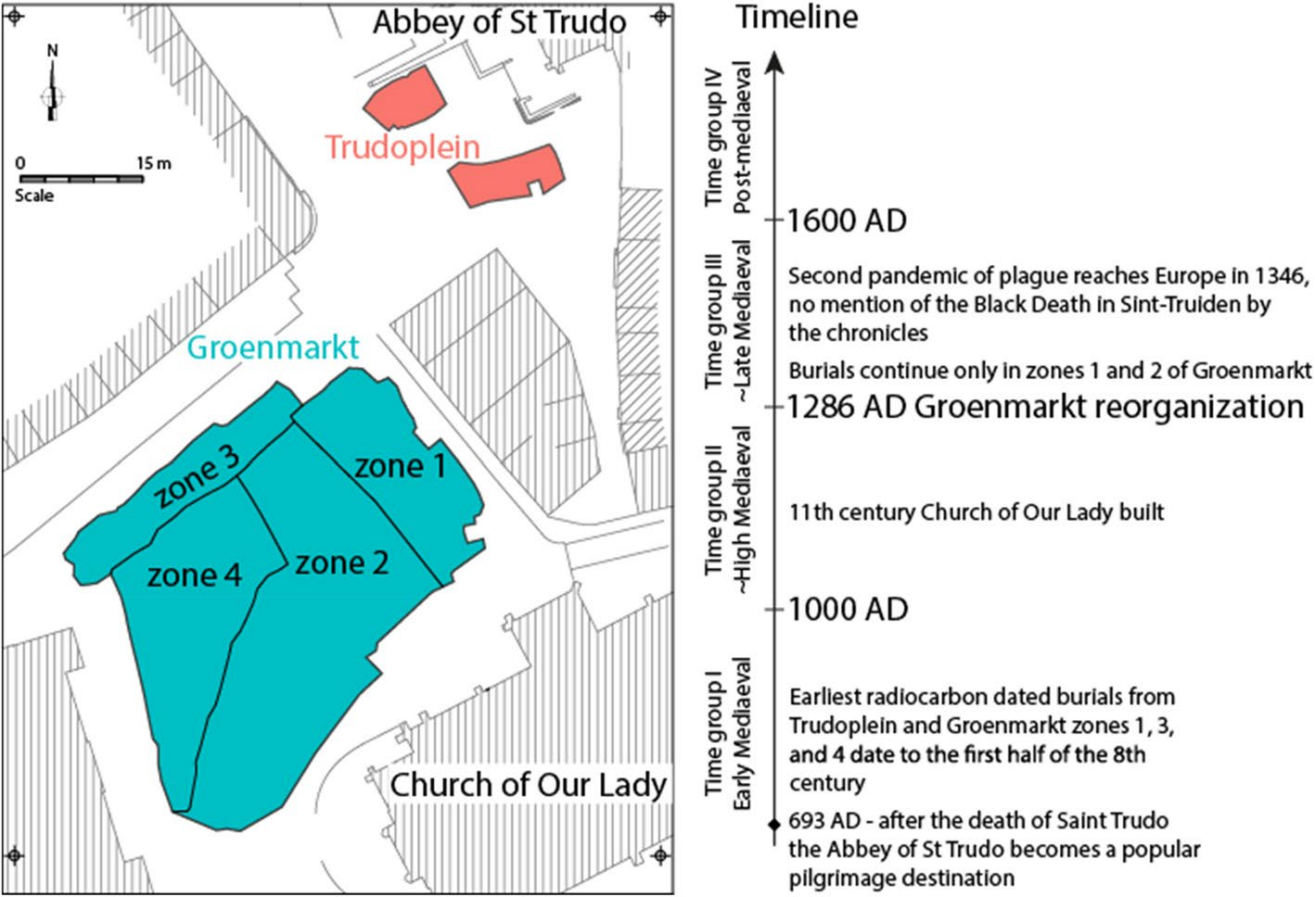
Sint-Truiden city center cemetery map. Two main burial groups at Trudoplein and Groenmarkt are highlighted by pink and green, respectively. Timeline of the main events mentioned in the text is shown to the right

A recent archeological excavation for the redevelopment of the city squares in the center of Sint-Truiden conducted between 2018 and 2020 unearthed a cemetery area that had been in use for over a thousand years, from the seventh c. until the eighteenth c. [18, 19]. The excavations were carried out at two adjacent locations (Fig. 1). The first—and the smallest—location was on the present-day Trudoplein square. This was part of a burial ground outside the abbey, near the tower of the Abbey’s church, used from the seventh to fourteenth century. The second location formed a large part of the present-day Groenmarkt square which was a burial ground from the seventh to the eighteenth c., extending before the eleventh c. possibly to the Trudoplein. From the eleventh to the eighteenth c., the eastern part of the Groenmarkt was the parish cemetery of Our Lady. From 1286, the burials continued only in zones 1 and 2, within the boundaries of the churchyard (Fig. 1). Over 3000 inhumation burials were uncovered, of which a subset of 404, selected by their skeletal completeness and availability of teeth, was included for ancient genomic analysis, representing a spatial distribution across the site and the broad temporal range. The availability of a large number of individuals from one site and the long use of the cemetery offers a unique opportunity to study the microgeography of the burials in time within a single urban site in relation to genetic ancestry, genetic relatedness, and metagenomic findings. The large number of genomes sequenced and the long-term use of the cemetery makes it also suitable as a model for the study of the development of a medieval European town, local population structure formation, and the influence of historically attested events, such as pandemics on genetic/population history and health.

## Results

We generated whole-genome shotgun sequence data from 404 human skeletal remains from Sint-Truiden, Belgium, dating from eighth to eighteenth c. AD, keeping for further analyses 372 genomes sequenced to mean coverage higher than 0.01x (median coverage 0.69x) and contamination rate < 0.05, including 332 with coverage > 0.1 × that were used in imputation-based analyses (Table 1, Additional file 1: Table S1). Radiocarbon dating and dietary isotope analyses of 114 individuals included in the genetic study, enabled probabilistic time group assignment of individuals by their burial context and showed a trend (*r* = 0.49) of higher δ15N values in time (Additional file 2: Fig. S1). Nitrogen isotope values reflect dietary behaviors, specifically related to protein intake, including terrestrial and aquatic protein-rich plants and animals [20]. The increasing δ15N values in time could either reflect rising marine fish consumption in inland Flanders in the late Middle Ages and in the post-medieval period [21] and/or generally improved nutritional status of the later phase individuals from the parish group. On the basis of radiocarbon dates and archeological context, the burials were assigned to broader time groups: I—700–1000 AD, Early Middle Ages; II—1000–1286 AD, High Middle Ages; III—1286–1600, later medieval; 1600–1800 AD, post-medieval (Additional file 1: Table S1). 1286 AD, as the time point separating time groups II and III, refers to the date at which there was a rearrangement of the current Groenmarkt [18]. Individuals from distinct burial zones (Fig. 1) and time groups were grouped together for downstream analyses.

**Table 1:** Ancient genomes analyzed in this study

			Number of individuals		
archaeological site, province, country	period, date range	source	total	≥0.01x	imputed
Sint-Truiden, Limburg, Belgium		1	404	372	332
	Time group I: Early Medieval, 700-1000		42	40	32
	Time group I/II		45	37	31
	Time group II: High Medieval, 1000-1286		207	191	171
	Time group III: later Medieval, 1286-1600		58	55	52
	Time group III/IV		22	21	19
	Time group IV: post-medieval, 1600+		30	28	27
Ypres, W-Flanders, Belgium	High Medieval	1	15	15	8
Koksijde, W-Flanders, Belgium	Merovingian, 7th to 8th cc	2			20
Wulpen, W-Flanders, Belgium	High to Late Medieval, 11th to 14th cc	2			5
France	Late Iron Age (LIA), 5th to 1st cc BC	3			18
England	Late Iron Age/Roman	4 to 6			30
England	Early Medieval, 5th to 9th cc	7			152
Ireland	Early to Late Medieval 7th to 13th cc	7			21
Netherlands	Early Medieval, 4th to 11th cc	7			22
Alt-Inden, N-Rhine, Germany	Merovingian, 5th to 8th cc	7			9
Lower Saxony, Germany	Early Medieval, 5th to 10th cc	7			26
Rathausmarkt, Schleswig, Germany	High Medieval, 11th to 12th cc	7			11
Denmark	High Medieval, 11th to 13th cc	7			7
Denmark	Viking Age, 8th to 11th cc	8			73
Norway	Viking Age, 9th to 11th cc	8			28
Orkney, Scotland	Viking Age, 9th to 11th cc	8			33

≥ 0.01 × —number of genomes sequenced to coverage ≥ 0.01 × and contamination rate < 0.05, imputed—number of genomes imputed in this study; all date ranges except for the Late Iron Age France are AD. Sources: 1—this study, 2—Sasso et al. 2024 [6], 3—Fischer et al. 2022 [22], 4—Scheib et al. 2023 [23], 5—Schiffels et al. 2016 [24], 6—Martiniano et al. 2016 [25], 7—Gretzinger et al. 2022 [5] and 8—Margaryan et al. 2020 [8]

In order to provide regional reference data from Sint-Truiden, we also sequenced the genomes of 15 individuals from the Hooge Siecken site in Ypres, in the modern province of West-Flanders, to coverage > 0.01x, including 8 to coverage > 0.1x (Table 1, Additional file 1: Table S1).

### Analyses of genetic ancestry and population structure

**Fig. 2:**
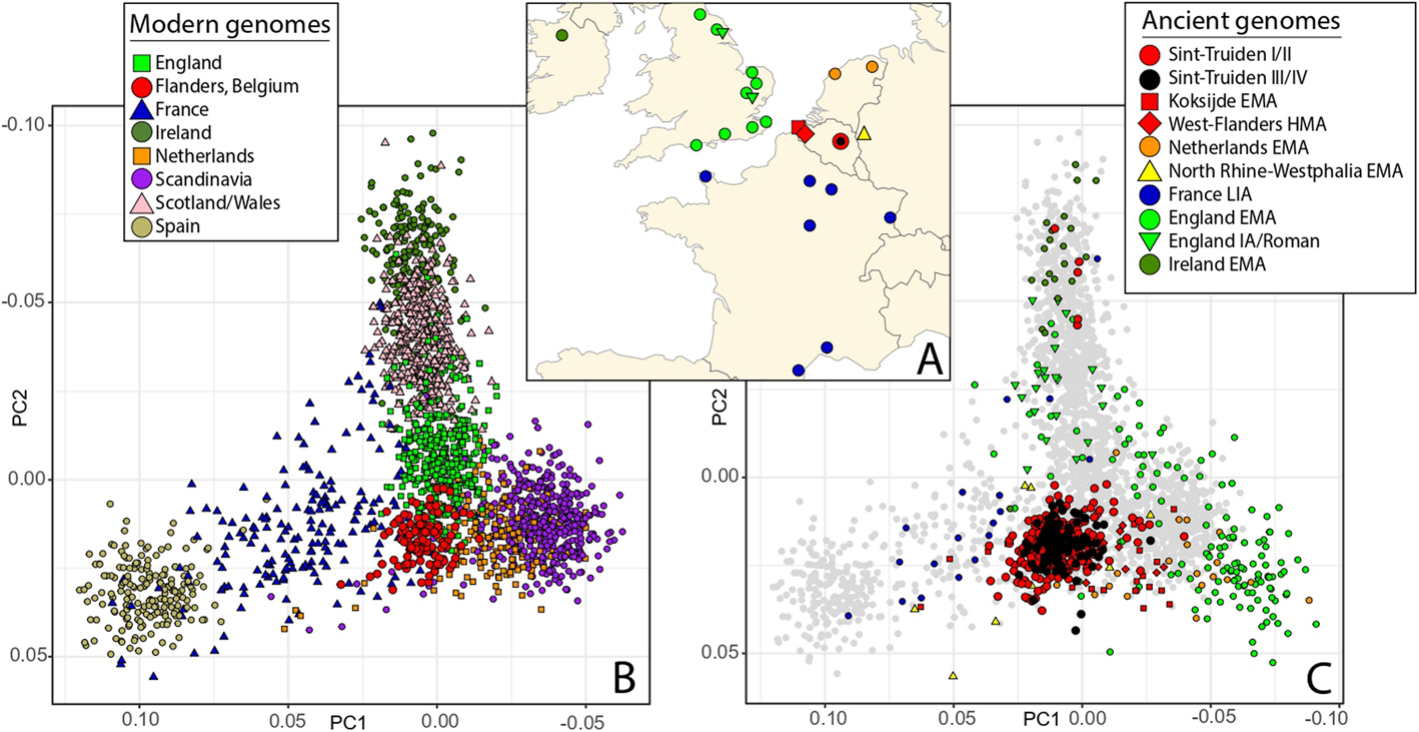
Sampling locations and genetic ancestry of the studied populations. **A** A map of the North Sea region with Early (EMA) and High (HMA) Middle Ages, Roman, and Late Iron Age (LIA) archeological sites used in data analyses, including genomes from Sint-Truiden (red), this study, and available reference data. PCA of selected modern (**B**) and ancient (**C**) genomes from Europe. PCA was run, after excluding closely related individuals, with FlashPCA2 without projection. **B** Modern population sources: 400 English, 191 French, 200 Irish, 190 Spanish, 443 Scandinavian (Danish, Norwegian, Swedish) and 400 Scottish/Welsh genomes from the UK Biobank; 112 Flemish and 195 Dutch genomes from the MinE consortium data [26, 27]. **C** 329 medieval/early modern imputed Sint-Truiden genomes and 8 Ypres individuals from this study, 20 Koksijde and 5 Wulpen individuals from Sasso et al. [6], 18 Iron Age French Gaul individuals from Fischer et al. 2022 [22], and 190 Early medieval genomes from Gretzinger et al. 2022 [5]

To explore the genetic ancestry of individuals buried at the cemetery of Sint-Truiden city center (Fig. 1), we first performed principal component analysis (PCA) on imputed genomes in the context of a broader reference set of genomic data. Analyses of ancient Sint-Truiden genomes in context of global reference populations showed that all individuals clustered tightly with those from present-day Northwest Europe (Additional file 2: Fig. S2). PCA focused on modern and ancient reference data from Northwest Europe (Fig. 2A) confirms the north–south structure observed previously in present-day data of the Low Countries [11], showing that the majority of the ancient Sint-Truiden individuals are placed on a PC1-defined cline between the Scandinavian and Dutch populations, on one end, and, modern and ancient genomes from France on the other (Fig. 2B, C). During the Early (EMA) and High (HMA) Middle Ages (time groups I–II), the Sint-Truiden population appears to have been more heterogeneous and spread out across this cline than it was during the later medieval and post-medieval period, being also more diverse in EMA/HMA than the whole Flemish population today (Fig. 2B). The second PC separates ancient and modern individuals from the British Isles from continental western Europe. Five individuals from the early (I/II) phase of Sint-Truiden appear as outliers by their PC2 values, clustering with the medieval and modern Irish and Scottish (Fig. 2B).

**Fig. 3:**
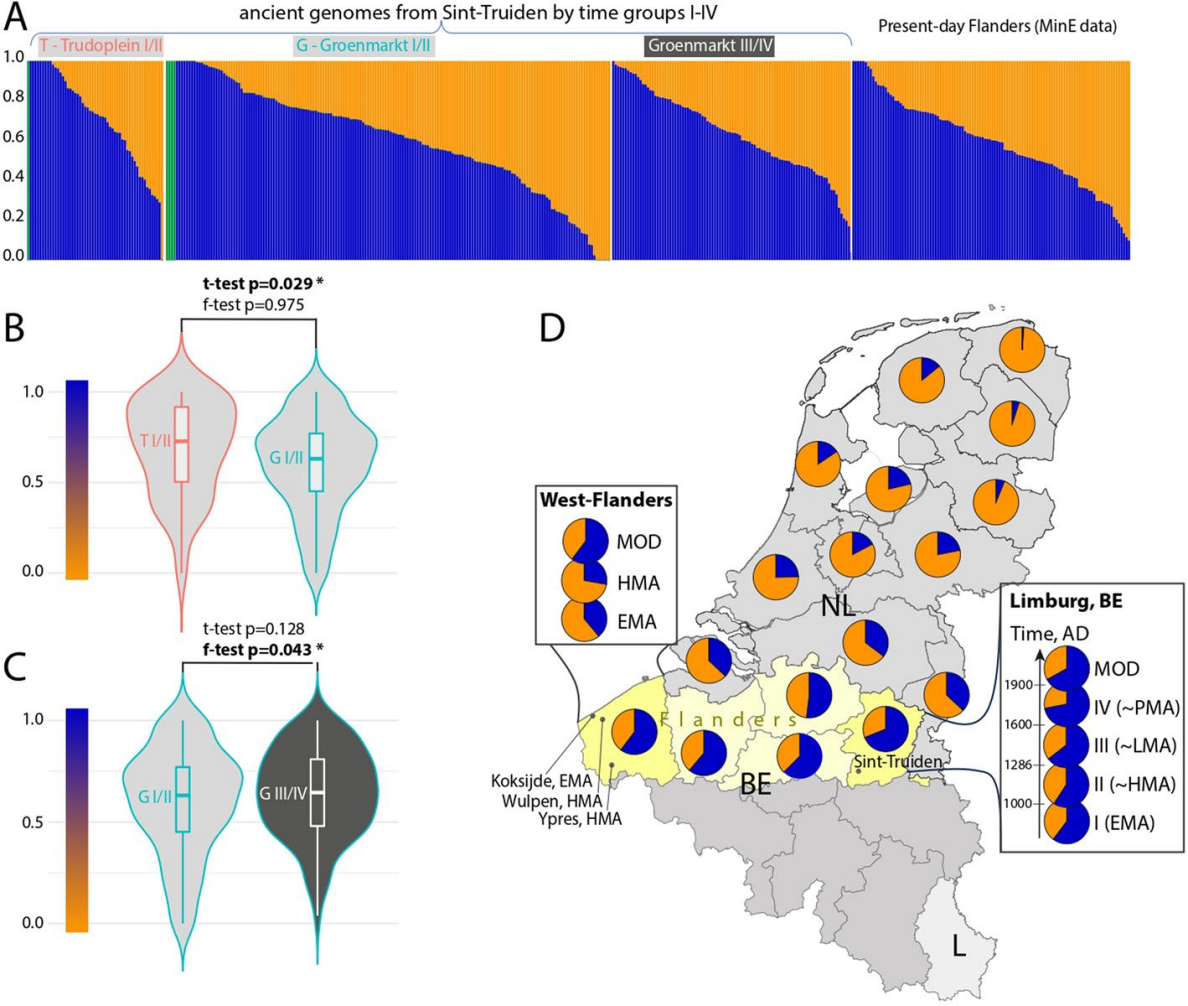
qpAdm based Gaulish and Germanic ancestry estimates in Flemish and Dutch provinces. **A** Presented Gaulish (blue) and Germanic (orange) ancestry proportions represent the best fit qpAdm model (Additional file 1: Table S3) for each population tested. **B** Violin plot comparing Gaulish ancestry proportions between Groenmarkt and Trudoplein burials. **C** Violin plot comparing Gaulish ancestry proportions between early (pre-1286 AD) and late (post-1286 AD) phase burials in Groenmarkt. Light gray background in panels **A–C** refers to early phase burials, predating 1286, and dark gray background to later phase burials postdating 1286. **D** Map showing average Gaulish (blue) and Germanic (orange) ancestry proportions by the provinces of the Netherlands and Flanders of Belgium. West-Flanders and Limburg provinces of present-day Flanders for which we present a transect of time data are highlighted with a darker yellow background. EMA—Early Middle Ages, HMA—High Middle Ages, LMA—Late Middle Ages, PMA—post-Middle-Ages, MOD—modern controls from the MinE project data

To further study the composition and temporal dynamics of the main ancestry components of the Sint-Truiden population, we used qpAdm [28]. As the PCA placed the majority of the Sint-Truiden genomes between genomes from Late Iron Age (LIA) France [22] and Early medieval (EMA) genomes from the Netherlands [5], we used these sources as proxies for Gaulish and Germanic ancestry, respectively. We found that people buried in Sint-Truiden had predominantly Gaulish ancestry (63% on average) since the Early Middle Ages with a minor (37%) Germanic component and that this ancestry composition has been retained for centuries largely unchanged, up to the population of present-day Limburg province of Flanders (Fig. 3, Additional file 1: Table S2). The higher Gaulish ancestry, ranging from 52 to 69% in different provinces of present-day Flanders, is part of a broader northeast to southwest cline of decreasing Germanic ancestry in the Low Countries [11]. In contrast to the transect of time in West Flanders, where the EMA and HMA genomes had lower (< 40%) than currently (> 60%) observed proportion of Gaulish ancestry, we find high temporal stability of the average Gaulish ancestry (60–72%) in the Sint-Truiden time series since the EMA period. Although we observe little change in the average ancestry composition, we find, consistent with the results of PCA, higher (*p* = 0.043, F-test) individual variance of ancestry in the Early/High Middle Ages than in the Late/Post-medieval period (Additional file 1: Table S3, Fig. 3). These results are also supported by analyses of supervised ADMIXTURE (*p* = 0.004, F-test), which reveal somewhat higher overall Gaulish ancestry (0.68 on average) across Sint-Truiden time groups than by qpAdm (Additional file 1: Table S2), and suggest that while the main ancestry sources were already in place in Sint-Truiden in EMA, the admixture process continued locally until at least the later medieval period. Considering the wide range of individual ancestry proportions in modern Flemish genomes (Fig. 3), this admixture process is likely still ongoing.

Two-way qpAdm admixture analyses of Sint-Truiden burials revealed a higher average proportion of Gaulish ancestry (71.9%, Fig. 3, Additional file 1: Table S2) in individuals buried in the Trudoplein near the St Trudo’s Abbey than in contemporary (time groups I and II) burials from the Groenmarkt (*p* = 0.029, 2-tailed *t*-test), supported also by the supervised ADMIXTURE results (*p* = 0.0006, 2-tailed *t*-test). Within Groenmarkt, we observed no difference in ancestry proportions between the burial zones or time groups (Figs. 1 and 3).

To test if the five Sint-Truiden samples that clustered with Irish and Scottish genomes on the PCA share allele frequency affinity specifically with any ancient or modern population, we applied outgroup f3-statistics on the five outliers and compared the results with the rest of the Sint-Truiden genomes (Additional file 1: Table S4). We found that the five outliers share more drift than the main group with Early Medieval Ireland and Viking Age Orkney than the remaining Sint-Truiden individuals. We found that both Viking Age Orkney and Early Medieval Ireland [5] can be used as ancestry proxies to model the outlier group, both with a two-way admixture model with Early Medieval (EMA) Netherlands as the alternative ancestry source. In a one-way admixture model scenario, with either Viking Age Orkney or Early Medieval Ireland as the only ancestry source, we could model the group as sharing 100% ancestry with Viking Age Orkney only. Importantly, such assignment does not indicate a direct link with the Viking Age Orkney population specifically, but confirms the placement within the PC showing similarity with genomes from Scotland/Ireland, and little to no Gaulish ancestry. At the individual level, all five outliers could be modeled as 100% derived from an ancient source in Scotland/Ireland, two of these outliers could also be described with 100% Germanic ancestry (Additional file 1: Table S5).

Analyses of mtDNA and Y chromosome haplogroups showed, similarly to autosomal PCA and qpAdm results, the predominance of west European ancestry components in Sint-Truiden (Additional file 1: Tables S1, S6-S7). Two individuals (ST1016 and ST2420) with mtDNA haplogroups typical to African populations (L2a and M1a) had no autosomal evidence of African ancestry and the observed mitochondrial sub-clades (L2a1k, formerly known as L2a1a, and M1a3a) appear to be found at low frequency today in Central and Southern Europe [29], reflecting likely prehistoric gene flow from Africa to Europe. We found no evidence (*p* = 0.38, 1-tailed *t*-test) of increased Germanic autosomal ancestry among 40 Sint-Truiden individuals with mtDNA haplogroups observed more commonly in Germanic source areas (match with > 2 individuals from EMA England, Netherlands, and Scandinavia) than in Celtic areas (match with < 2 individuals from Bronze/Iron Age France, Netherlands, or Britain, Additional file 1: Table S6). The basal Y-haplogroup frequencies of EMA and HMA Sint-Truiden were similar to those of populations from surrounding regions and have remained relatively unchanged in modern Flemish population [30], with no specific outliers indicative of inter-continental migration (Additional file 1: Table S7, Additional file 2: Fig. S3). All four male outliers identified with PCA belonged to the R1b2-L21 (R1b1a1b1a1a2c1a) clade (Additional file 2: Fig. S4) that was uniquely frequent in Bronze and Iron Age Britain [31], being still the most common haplogroup found in present-day Scotland and Ireland. As the four outliers belong to distinct subclades of R1b2-L21 they are not closely related to each other in their patrilines.

**Fig. 4:**
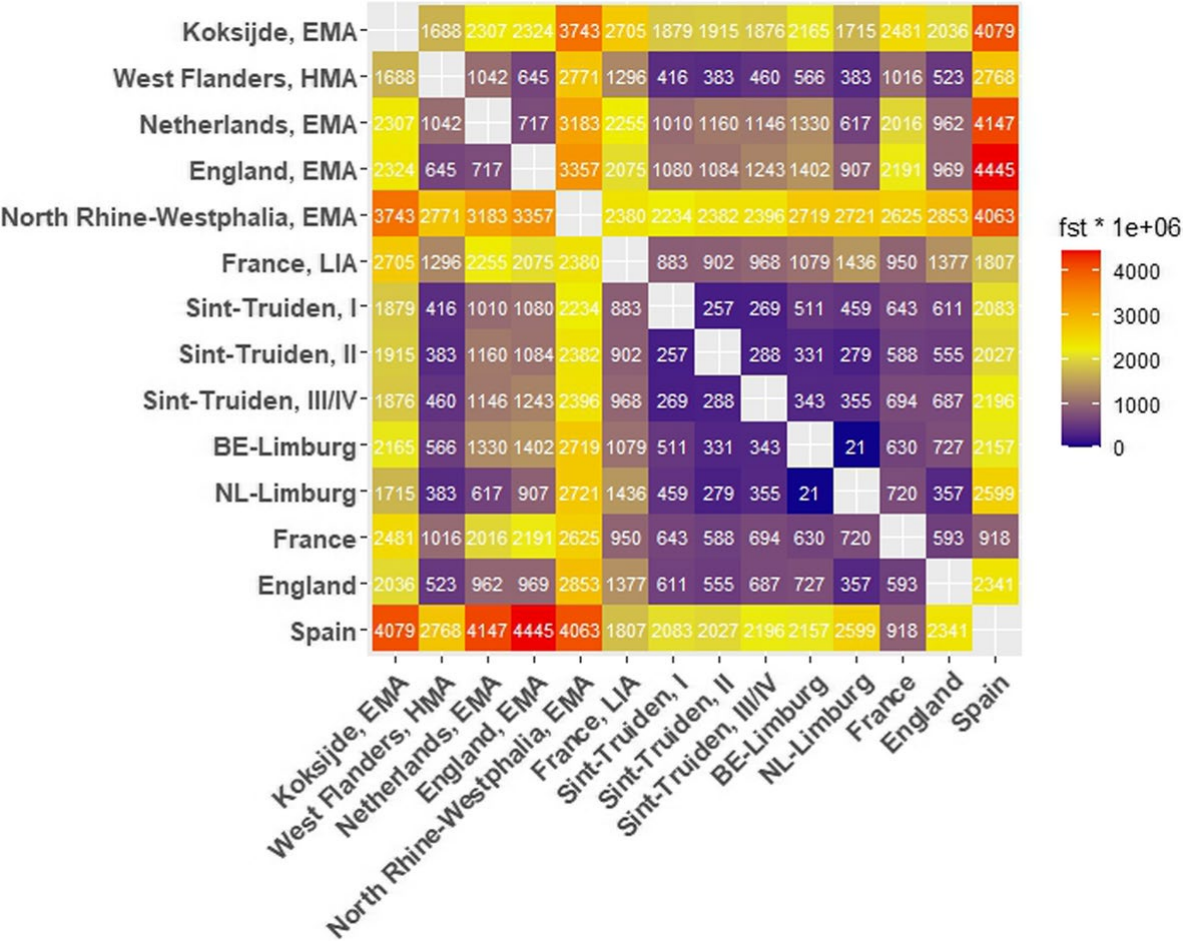
Genetic distances between the ancient and modern populations of the region. Genetic distances are presented as Fst × 1000000. Sint-Truiden time groups I = 675–999 AD (~ EMA), II = 1000–1286 (~ HMA), III/IV = 1287–1775, LIA = Late Iron Age, EMA = Early Middle Ages, HMA = High Middle Ages. BE-Limburg = Belgian Limburg, NL-Limburg = Dutch Limburg. Data sources: Sint-Truiden, this study; Koksijde, Sasso et al. [6]; West-Flanders, this study and Sasso et al. [6]; Early Medieval Netherlands, England and North Rhine-Westphalia, Germany – Gretzinger et al. 2022 [5]; Late Iron Age (LIA) France, Fischer et al. 2022 [22]; present-day Belgian and Dutch Limburg, the MiNE Consortium data [26, 27]; France, England and Spain, the UK Biobank data [32]

A previous study by Byrne et al. [11] had observed high levels of genomic differentiation between regional groups in the present-day Netherlands, some of the genetic distances between local groups being greater than differences between distant regional populations of present-day Europe. To assess regional continuity over time and compare the extent of modern population stratification with the extent of regional genetic differentiation in medieval Flanders, we measured genetic distances between the ancient and modern populations from the Low Countries and West Europe with Fst and found that the genetic distance between the Sint-Truiden population and the population of Merovingian Koksijde in the province of West Flanders in the first millennium (EMA period) as well as that of a Merovingian site in North Rhine-Westphalia in Germany were nearly as high as the distance between modern Belgian Limburg and Spain (Fig. 4), while the distance with the early medieval populations of The Netherlands and England was smaller. However, by the HMA period, the Fst differences between Sint-Truiden and the two high medieval populations from West Flanders had become similarly low and comparable to the distances among the modern populations of all Flemish provinces. The genetic distances between present-day Limburg and the HMA West Flanders, on the one hand, and the Sint-Truiden population sampled at different time points, on the other, remained low over time, suggesting that the unusually high allele frequency differentiation in the EMA period is likely due to the atypical ancestry profile of the Merovingian Koksijde community [6].

**Fig. 5:**
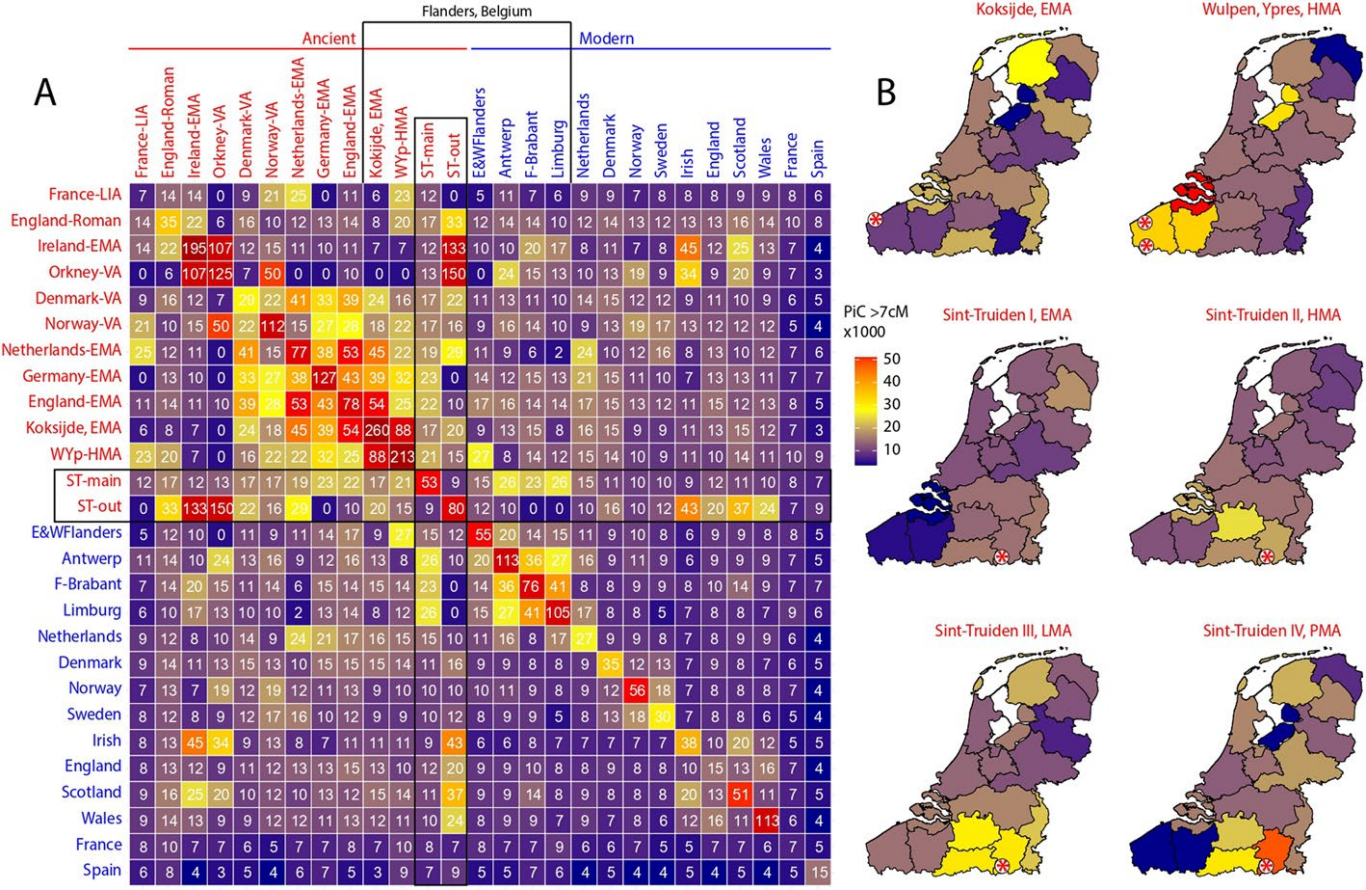
Probability of individual connectedness (PiC) with modern and ancient populations. **A** Heatmap of probabilities (× 1000) of between individual sharing of > 7 cM segments within and among populations. Ancient population data (shown red) include data for Sint-Truiden and Ypres from this study, Early medieval (EMA) data from Gretzinger et al. 2022 [5], Sasso et al. [6], Late Iron Age (LIA) genomes from Fischer et al. 2022 [22], Viking Age (VA) data from Margaryan et al. 2020 [8], and Roman period data from Scheib et al. 2023 [23], Martiniano et al. 2016 [25] and Schiffels et al. 2016 [24]. Modern reference data include populations from the UK Biobank [32] and MinE Project data [26, 27]. ST-main – 318 genomes from the Sint-Truiden main group; ST-out—five outliers identified by PCA (Fig. 2). B. PiC scores estimated between present-day provinces of the Netherlands and Belgium, MinE project data [26, 27] and transect of time data from West Flanders ([6], this study) and Sint-Truiden (this study). Red stars on each map indicate the geographic location of the archeological sites with data

To further study the spatial and temporal dynamics of regional population structure in the Low Countries in the last 2000 years, we estimated the probabilities of inter-individual sharing of > 7 cM IBD segments for Sint-Truiden and reference genomes with IBIS [33]. We found contrasting patterns of IBD sharing for the Sint-Truiden main group of individuals and the five outliers identified with PCA (Fig. 5A). The main group shows the highest affinity to present-day individuals from the Belgian provinces of Limburg, Antwerp, and Flemish Brabant and to Early Medieval genomes from Germany, the Netherlands, and England. The outliers, in contrast, show on average low sharing probabilities with these groups and instead show the highest affinity to Early Medieval and modern populations from Scotland and Ireland. Notably, the average probability of finding individual pairs sharing > 7 cM IBD segments among the Sint-Truiden main group of burials (0.053) is lower than in sets of MinE consortium [26, 27] controls whose four grandparents were born in present-day Limburg (0.105) or Flemish Brabant (0.076) (Fig. 5A). This suggests genetic heterogeneity of the main group of burials beyond the identified five outliers. The comparative analysis of IBD sharing probabilities between genomes from present-day provinces of the Netherlands and Belgium on the one hand, and ancient genomes sampled at different time points in West Flanders and Limburg provinces of Belgium on the other (Fig. 5B) revealed that EMA genomes from Koksijde and Sint-Truiden had only broad geographic affinity to present-day Low Countries and that the finer regional province-specific patterns of interindividual connectedness emerged from HMA onwards, with the post-medieval (time group IV) population of Sint-Truiden showing clearly the highest IBD sharing probability with the present-day genomes from its surrounding Belgian Limburg province.

### Genetic relatedness analysis

**Fig. 6:**
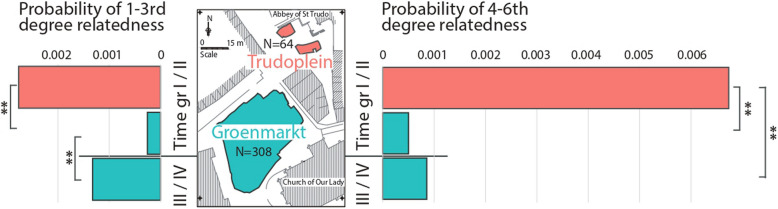
Probabilities to observe genetic relatedness between Sint-Truiden burials. The probabilities of 1st–3rd degree relatedness are expressed as the ratio of observed relationships in the burial place and time with KIN and READ2 (Additional file 1: Table S8) and the total number of individual pairs with minimum aggregate coverage to detect them. The probability of 4–6th degree relatedness is expressed as the ratio of observed relationships with IBIS (Additional file 1: Table S9) and the total number of pairs of imputed individual genomes available from the burial place and time for the analyses

High rates of genetic relatedness in a sample can influence the results of population genetic analyses but they can also be informative of demographic and social aspects of the burial population. Bigger communities or those with high rates of immigration would be expected to show lower rates of relatedness than local communities with small population size. High proportion of inter-individual relationships in cemeteries can be expected if the burial ground was used by the same small community continuously over a period of time. To determine the extent of genetic relatedness among the Sint-Truiden burials, we first performed a screening of closely related pairs in 372 genomes with coverage > 0.01 × with KIN [34] and READ2 [35]. Among the 70,790 individual pairs with more than 30,000 overlapping SNPs, we detected 17 cases of 1st–3rd degree relatedness (Additional file 1: Table S8), including six pairs with 1st degree relationships and five with 2nd degree relationships. The average probability of 1st–3rd degree relatedness (0.00024) of the entire set of Sint-Truiden burials is 79-fold lower (*p* < 0.00001) than the rate observed in medieval parish cemeteries in Cambridge [9]. A similarly low rate (0.00023) is observed among the 12,936 pairs of the biggest subset of time group II (1000–1286 AD) burials from the Groenmarkt. We find, however, notable differences in the rates of close degree relationships between locations and time periods: in the early phase (before 1286, time groups I and II), Trudoplein burials show a more than tenfold higher rate than Groenmarkt burials of 1st–3rd degree relationships, and time group III and IV burials in Groenmarkt show a more than fivefold higher rate than earlier burials in the same location (Fig. 6). These results of relatively higher rates of genetic relatedness in Trudoplein and later burials of Groenmarkt than among the earlier burials of Groenmarkt are further corroborated by the screens of cases of 1st–6th degree relatedness (Fig. 6 right) with IBIS [33]. We observed a 13-fold higher rate of 4–6th degree relationships among Trudoplein than among Groenmarkt burials. The low probability of 4–6th degree relatedness in time group I and II burials of Groenmarkt (0.0005) is comparable to the rate (0.0003) observed in 183 MinE consortium controls sampled across Belgium while being lower than the rate within individual provinces. The 99 individuals with at least one 1st–6th degree relationship show higher (*p* = 0.005, 2-tailed *t*-test) proportion of Gaulish ancestry (0.68) than individuals with no relationship detected (0.60). This difference, however, is linked with the higher Gaulish ancestry in the Trudoplein group as the proportion of Gaulish ancestry in Groenmarkt individuals (0.65) is not significantly (*p* = 0.11) higher than observed in Groenmarkt individuals with no relationships (0.60).

Low rate of genetic genetic relatedness among Groenmarkt burials could reflect either large effective population size or large catchment area of rural to urban migration potentially including migrants from sources with low effective population size. To distinguish between these possibilities we screened the Sint-Truiden genomes for evidence of runs of homozygosity (ROH), which are indicative of parental relatedness, and tested the relationship between heterozygosity at variants with MAF > 0.05 and genetic ancestry. Using the pseudo-haploid model of hapROH [36], we find high correlation (*r* = 0.99991) between imputed and non-imputed pseudo-haplodized results for ROH segments exceeding 8 cM (Additional file 2: Fig. S5, Additional file 1: Table S10), which encourages us to use imputation-based results with more individuals. Our ROH data provides evidence for 14 individuals with ROH > 8 cM, interestingly all of which were buried in Groenmarkt. Overall, the prevalence of individuals with ROH > 8 cM tracks in Sint-Truiden (~ 4%) is similar to the ~ 5% rate observed in late medieval Cambridge [9]. None of the individuals in our dataset showed evidence of “long ROH,” the sum of ROH > 20 cM lengths exceeding 50 cM [36], a threshold typically indicative of inbreeding between first or second cousins. However, we do find two individuals (ST1186 and ST1233) with ROH > 20 cM, suggesting parental relatedness at the level of fourth cousins or closer [37]. Notably, one of these individuals (ST1233) is among the five outliers with Scottish or Irish ancestry. We find a weakly positive correlation between heterozygosity and Gaulish ancestry, which is stronger (*r* = 0.28) in individuals from Trudoplein (Additional file 2: Fig. S6). In combination with the differences in genetic relatedness probabilities, these results suggest that the catchment area of the Groenmarkt burials was different from Trudoplein burials, likely including different external communities.

The observed low rate of relatedness is notable also in light of the presence of at least 80 multiple burials, one from Trudoplein and all others from the Groenmarkt, of which twelve graves included multiple individuals whose genome we had sequenced. Not a single case of 1st–7th degree relatedness was detected between individuals (including child–adult pairs) co-buried in 12 graves from which we had sampled multiple individuals (Additional file 1: Table S11) suggesting that time of death may have been more important than genetic relatedness in defining co-burials. On the other hand, we are likely to underestimate genetic relatedness, particularly in co-burials with children, given our low rate of success of retrieving DNA from the teeth of sub-adults. However, we do find a pair of 3rd degree related adult males (ST2000 and ST2228) with identical mitochondrial DNA buried in different graves with unrelated individuals in a co-burial context, both in zone 2 and layer 7 in Groenmarkt. Furthermore, six of the individuals from co-burials had a distant 4–6th degree relationship with individuals from single burials, including ST2000 who had four such relationships. While the higher concentration of genetic relatedness findings in Trudoplein could signify the importance of the proximity of the Abbey for selected groups of families of the city, the sporadic cases of genetic relatedness between but not within Groenmarkt co-burials further confirm that genetic relatedness did not play a primary role in determining where precisely in the cemetery individuals were buried.

To specify autosomally detected genetic relatedness relationships and to explore sex-specific differences in relatedness, we determined which pairs shared mtDNA and Y chromosome haplogroups. Analyses of mtDNA revealed 248 distinct haplogroups, including 171 detected in only a single individual, and 77 haplogroups shared by 214 individuals (Additional file 1: Tables S1, S6). Among the 332 individuals that were included in IBD analyses, only seven pairs of individuals out of 158 with shared mtDNA haplotype showed evidence of autosomal relatedness. In the majority of the cases (151 pairs, 95.6%) sharing of mtDNA haplotype was not indicative of 1st–6th degree of relatedness. Unsurprisingly, considering the decreasing likelihood to share either matrilineal or patrilineal relatives with increasing degree of relatedness, we found the majority of the matches, five in mtDNA and one in Y chromosome, among the nine cases of the first and second degree pairs (Additional file 1: Table S9). In contrast, among the 64 pairs of 3rd–6th degree, only two mtDNA and a single Y chromosome match was detected. We observed no sex difference (*p* = 0.22, 2-tailed *t*-test) in the number of relationships among the 99 individuals identified with 1st–6th degree relationships: 51 were male including 12 with multiple 1st–6th degree relationships in the data, while among 48 females we found 17 individuals with multiple relationships.

### Changes in effective population size

**Fig. 7:**
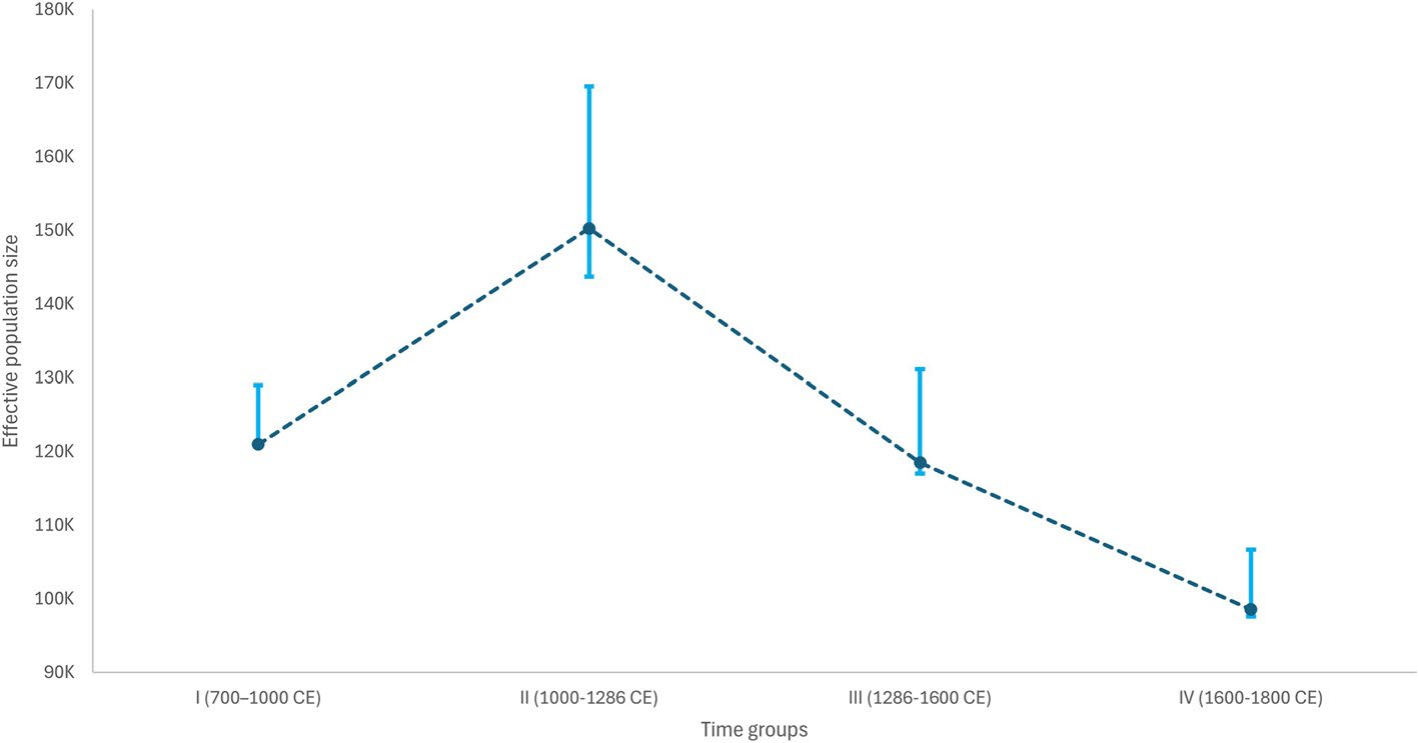
Effective population size (Ne) over time. Effective population size (Ne) of the Sint-Truiden city center population between the eighth and the eighteenth century inferred by plotting the mean Ne estimated for each time group over its past 4 generations. The dark blue dotted line joins the different mean Ne estimates and the 95% confidence intervals obtained with bootstrap quantiles are represented by the light blue error bars

Effective population size is often estimated from genetic data as a proxy of the number of individuals involved in reproduction, being widely used for inferences about the demographic, social, and cultural history of populations [38, 39]. To determine that of the Sint-Truiden city center population, we independently ran the software HapNe-LD [40] on each time group, removing closely related individuals and the five outliers identified by PCA (Methods). With its regularization mechanism, HapNe avoids spurious inferences of demographic fluctuations and favors smoother curves instead. As foreseen by the authors of the method in the case of insufficient demographic signal in the data, effective population size was thus always inferred to be constant (Additional file 2: Fig. S7). To get around this limitation, we plotted each time group’s mean Ne over the past 4 generations in order to obtain a broad overview of changes in effective population size between the eighth and eighteenth centuries (Fig. 7). We note an increase in effective population size from the time groups I to II, followed by a drop reaching its minimum with time group IV. This tendency is similar to the inferred historical Ne trajectory Byrne et al. [11] obtained using modern DNA from various Dutch provinces, including that of Limburg. Our results also corroborate the higher diversity noted in the earlier periods through PCA (Fig. 2) and qpAdm (Additional file 1: Table S3, Fig. 3).

### Health and phenotype-informative variation and metagenomic findings

The study of the Early Medieval site Koksijde in Belgium revealed major allele frequency differences between groups of Gaulish and Germanic ancestry in variants associated with pigmentation and dietary phenotypes [6]. Firstly, to gain broader insights into phenotype-related changes through time, we focused on a shortlist of 112 diet, immunity, and pigmentation-related variants including those previously highlighted as targets of selection (Additional file 1: Table S12). We compared the allele frequencies of these variants in Early/High medieval Sint-Truiden with modern Flemish genomes from the MinE dataset [26, 27]. Similarly to the results of Sasso et al. [6], none of the examined variants retained significance after multiple test corrections (Additional file 1: Tables S7, S12–S13). These results reflect the stability of average allele frequencies through time, observed in our genome-wide analyses (Fig. 3). While we found no evidence of major temporal changes in tested phenotype-informative markers, we observed significant differences related to ancestry. We found that individuals carrying at least one of the five red hair causing alleles in the *MCR1* gene had higher proportion of Germanic ancestry, while individuals carrying the rs7944926-G allele in the *DHCR7* gene, which is associated with higher levels of vitamin D precursor 25(OH)D3 (calcidiol) in the blood [41], had higher proportion of Gaulish ancestry (Additional file 2: Fig. S8). Notably, the increased 25(OH)D3 levels have also been observed in redheaded people [42].

#### Variation in immunity genes

The second pandemic of plague with its high mortality is likely to have affected allele frequencies of immune genes associated with infectious disease vulnerability. Reports of significant enrichment of high Fst variants among immunity genes in comparisons of pre- and post-Black Death cohorts [43] have been contested by subsequent studies [9, 44]. Our estimates of Fst between 239 early (time groups I and II) and 46 late (time groups III/IV and IV) phase imputed Sint-Truiden genomes revealed no enrichment of highly differentiated (*F*_*ST*_ > 99th percentile) variants related to innate immunity either before or after the LD pruning step (Additional file 1: Table S14). We observed a cluster of highly differentiated variants in the 22:22,481,208–22,725,114 region of the *IGL* locus behind the enrichment signal of highly differentiated variants related to adaptive immunity, which disappeared after the LD pruning step (Additional file 1: Table S14).

#### Genome-wide screens of Fst outliers

Despite not finding significant enrichment signals of immunity, we carried out a systematic screening of the genome for variants that most highly differentiated between the early and late phase genomes. Our genome-wide screens of 37,403 independent SNPs retained after LD pruning revealed 17 variants as Fst outliers in all comparisons made between the early (time group II) and later phase time groups (Additional file 2: Fig. S9). Eight of these variants map to genic regions and are all intronic variants (Additional file 1: Table S15). The variant with the highest average Fst among all groups is located in the gene *PLCE1* (Additional file 2: Fig. S10), involved in T-cell migration to inflamed skin [45] and mediating macrophage activation [46]. The gene itself is associated with several phenotypes including leukocyte and monocyte counts [47].

#### Metagenomic analyses

The metagenomic screening of 372 Sint-Truiden genomes for reads matching human-associated microbial pathogens revealed probable findings in 35 individuals. In most cases, identifications are based on low levels of DNA and can thus not be fully validated (Additional file 1: Table S16). The findings included 10 individuals with likely cases of hepatitis B virus (HBV), five with *Yersinia pestis*, six with *Borrelia recurrentis*, three with human betaherpesvirus 6A (HHV-6A), one with human betaherpesvirus 6B (HHV-6B), three with *Leptospira interrogans*, and two with herpes simplex virus 1 (HSV-1) as well as eight other viral or bacterial findings in single individuals (Additional file 1: Table S16). One individual (ST1319) showed evidence for co-infections of *Yersinia pestis* and HBV. This is in line with previous studies reporting the presence of *Y. pestis* with a chronic infection [44, 45]. Capture-based analyses of individuals with *Y. pestis* reads failed to produce enough sequence coverage for strain-specific assignments in six individuals while in case of SK1516 two substitutions were detected that are not found in Black Death strains while present in all strains in the main Second Plague Pandemic lineage (Supplementary Information, Additional file 1: Tables S17-18, Additional file 2: Fig. S11).

**Fig. 8:**
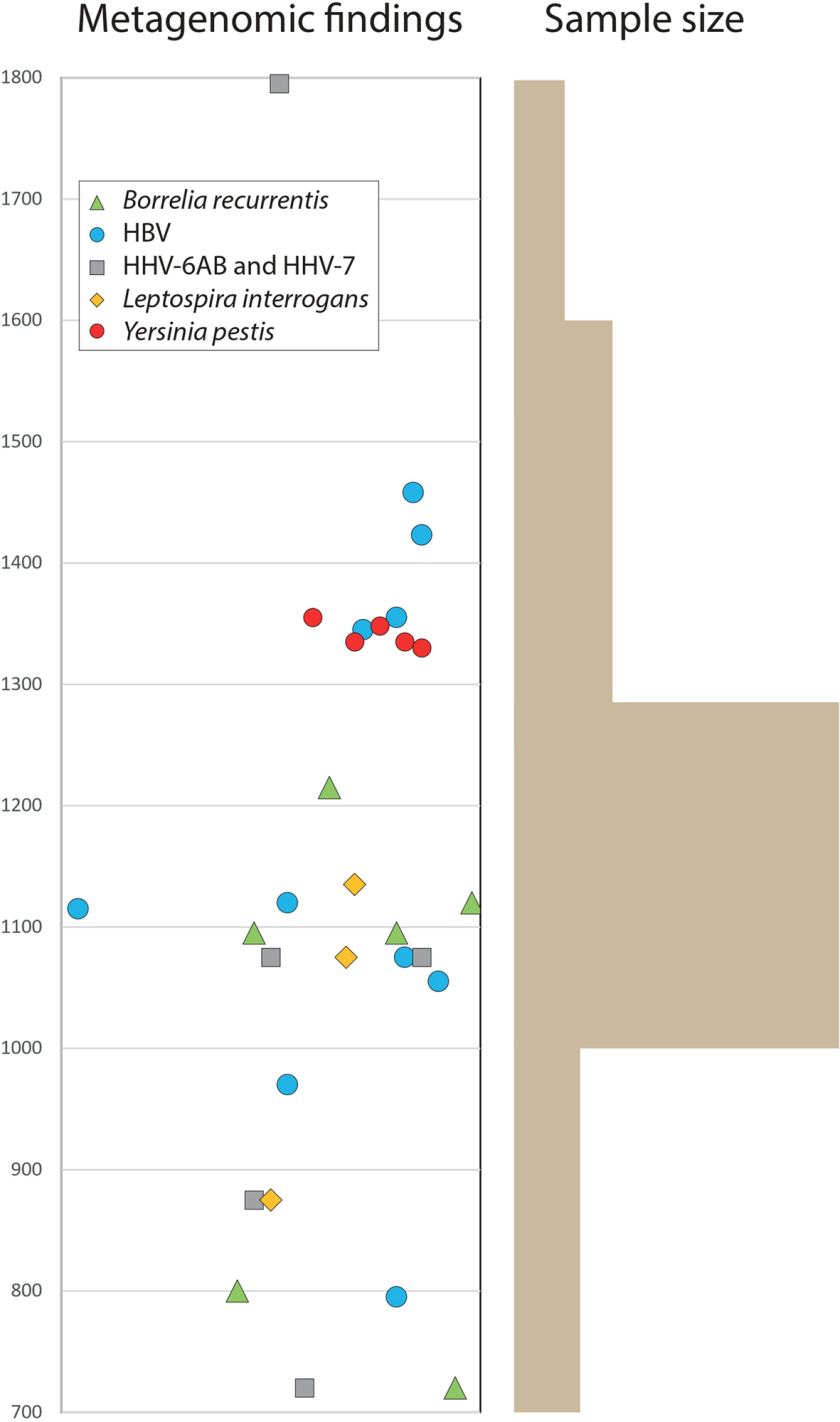
Temporal distribution of the metagenomic findings. Individuals with pathogen findings and radiocarbon dates are presented for the subset of pathogens detected at least twice (Additional file 1: Table S16). HBV—hepatitis B virus, HHV—human betaherpesviruses. The mean value of the 95% range of the radiocarbon date of each individual is shown on the *y* axis, *x* axis values showing δ15N values

Further, more detailed strain-level analyses of full HHV-6AB genomes findings will be presented in a separate study [46]. Radiocarbon dating of 34 individuals with pathogen findings showed a wide temporal range for most pathogens, except for *Y. pestis* findings, all of which dated to the fourteenth c. (Fig. 8). Two adult males with *Y. pestis* findings, buried apart in different burial zones of the Groenmarkt, were found to be second degree related and to share the same mtDNA haplogroup.

## Discussion

### Homogenization during urbanization

Our analyses of genetic variation in Sint-Truiden population over a time transect spanning more than a thousand years revealed a process of homogenization of two distinct genetic ancestries during the intensive urbanization phase in the Low Countries. In the Early and High Middle Ages, the population of Sint-Truiden was more heterogeneous, with approximately 10% of the individuals clustering outside the range of the Late/Post medieval and modern genomes from Flanders in the PCA (Fig. 2). This indicates that the genetic diversity of the EMA/HMA population of Sint-Truiden was remarkably higher than the modern genomes from present-day Flanders, a pattern also observed in the EMA population of Koksijde [6]. Moreover, the genetic differences between Sint-Truiden and Koksijde during the EMA were larger than those between present-day Spain and Flanders (Fig. 4). Over time, the genetic variation decreased, and the ancient genomes increasingly resembled the present-day population in the region around Sint-Truiden (Fig. 5).

Contrary to what might be expected based on the abbey’s international connections, as noted in its chronicles [15, 48, 49], and the long-distance trade of products from Sint-Truiden, no long-distance migrants from regions outside Northwest Europe were identified among the genomes we studied. Most individuals in Sint-Truiden, based on their clustering with modern-day genomes from Flanders, likely had local origins in the region surrounding Sint-Truiden. This contrasts with findings from the late medieval Sint-Rombout's parish cemetery in the city Mechelen, 54 km from Sint-Truiden, where several individuals within a small sample showed Mediterranean ancestry [50]. The only exception in our sample to the predominantly local ancestry profile typical to the Low Countries is a distinct group of five individuals with profiles similar to those from Ireland and Scotland (Figs. 2 and 5). Four of the outliers date to 1000–1286 and one to Early Middle Ages, and they were buried separately at the cemetery site. All male individuals belonged to the Y chromosome haplogroup R1b2-L21 clade, whose ancestry is uniquely related to the British Isles [31]. Despite two of the outliers being juveniles, none of the individuals shared close genetic relationships with each other or anyone else sampled from Sint-Truiden, and all four Y chromosomes belonged to different subclades, ruling out close patrilineal relatedness. It is plausible that these persons were connected to the Benedictine abbey, although the abbey’s chronicles make no mention of connections to Ireland or Scotland [15, 48, 49]. Therefore, they may have been specialized craftsmen or pilgrims during the abbey’s period of growth when a large church and other parts of the abbey were constructed [51].

### Admixture of Gaulish versus Germanic ancestry

Amidst the higher inter-individual diversity observed in the EMA and HMA, we noticed temporal stability in the main ancestry components in Sint-Truiden over the span of a thousand years, with higher Gaulish and lower Germanic ancestry based on the qpAdm analysis (Fig. 3). These ancestry components were present from at least the Early Medieval period and did not show notable changes over time in Sint-Truiden and the surrounding province of Limburg. This suggests that the admixture of Germanic and Gaulish ancestries was a prolonged process over centuries, rather than an instant event. Consequently, the admixture dates, e.g., as estimated by Byrne et al. [11], could potentially be separated from the initial migration events that led to the geographic distribution of ancestry components by a considerable amount of time.

The prolonged nature of Germanic and Gaulish admixture is particularly evident in West-Flanders. Our results indicate that Germanic ancestry was the dominant component in EMA and HMA, while Gaulish ancestry is more prevalent in the current population (Fig. 3). This suggests that, in contrast to Sint-Truiden and Limburg, the population composition in West-Flanders underwent structural changes in the post-medieval period. One explanation could be the extensive migration event at the end of the sixteenth c. from northern France—presumed to have higher Gaulish ancestry—to West- and East-Flanders after the strong population reduction related to the Eighty Years’ War. It is estimated that around 20% of the West-Flemish population in the early secenteenth c. had recent origins in current-day France, a finding supported by Y-chromosomal research [52].

Interestingly, the detailed understanding of the temporal aspects of the admixture of Germanic and Gaulish ancestries also explains the genetic cline observed across the Low Countries, as described for the first time by [53] and [54]. Byrne et al. [11] dated the primary admixture event in the Netherlands, which underlies this cline, to the eleventh century. As Byrne et al. [11] observed lower effective population size in the northern provinces of the Netherlands they suggested that the ancestry cline may have been caused by population expansion from south to north. Our ancient DNA analysis suggests that the origins of this admixture process, rather than event, are even older, possibly dating back to the start of the expansion of people with higher Germanic ancestry from the north of this region at the end of the Roman Period (Fig. 3). This demonstrates that ancient DNA analysis can elucidate current fine-scale geographically correlated components. Moreover, our results for Sint-Truiden illustrate the biological relevance of taking into account these ancestry components in phenotype analyses. We found in medieval Sint-Truiden that individuals carrying at least one of the five red hair-causing alleles in the *MCR1* gene had a higher proportion of Germanic ancestry, while individuals carrying the rs7944926-G allele in the *DHCR7* gene, which similarly to red hair causing mutations [42] is associated with higher levels of vitamin D precursor 25(OH)D3 (calcidiol) in the blood [41], had a higher proportion of Gaulish ancestry.

### Ancestry differences and relatedness patterns within Sint-Truiden city center by space and time

Within the archeological site in the city center of Sint-Truiden, we observed higher Gaulish ancestry in the genomes of individuals buried in the present-day Trudoplein compared to those buried at the present-day Groenmarkt (Fig. 3). The area in the Trudoplein was used between the seventh and fourteenth centuries. Due to its proximity to the former St Trudo’s Abbey, it is possible that individuals buried at the Trudoplein were more closely connected to the Abbey, either through services provided to the Abbey or through their origins, while those buried at the Groenmarkt before 1286 may have been local townsfolk or pilgrims. Saint Trudo donated Sarchinium and the abbey to the Bishop of Metz, a city in current-day France, and the Bishop of Metz remained the abbey’s authority, as well as over half the town, until 1227 [51]. One hypothesis is that those buried near the abbey were connected to Metz, where Gaulish ancestry is believed to be more prevalent. The abbey chronicle says that in 1083–1085, which falls in the period in which most of the individuals with higher Gaulish ancestry have been dated, abbot Lanzo repopulated the abbey with monks from the Metz region ([49], pp. 29–30). Moreover, we also observed significant differences in rates of close (1st–3rd degree) and medium (4–6th degree) relatedness within the cemetery, with more relatedness among the individuals buried at Trudoplein than at Groenmarkt (Fig. 6). This suggests that the individuals buried in the two excavated cemetery areas in the city center of Sint-Truiden had different social and/or ancestry backgrounds, demonstrating a more complex necrogeography of the archeological site than previously expected without genetic data [18]. We acknowledge the fact that inference of more distant degrees of relatedness is less accurate with rates of correct classification dropping from 80% for the 4th degree to 58% for the 6th degree with the IBIS method we use [33]. However, most incorrect classifications (> 98% in case of the 5th degree) are expected to fall within + / − 1 degree [33]. We expect, thus, that our 4–6th degree inferences are relatively robust as indicators of medium relatedness and should be interpreted with the + / − 1 degree assignment error rather than to be taken at their face value. Furthermore, given similar proportions of Gaulish and Germanic ancestry in both groups, we do not expect the differences in ratios of intensity of relatedness observed for Trudoplein and Groenmarkt to be strongly influenced by ancestry differences.

Our analyses of effective population size (Ne) changes revealed about a 30% drop of Ne after 1286 AD. Many factors may have contributed to this change. Considering the low rates of genetic relatedness ties observed, it is possible that the Ne values are affected by temporary differences in the proportion of individuals (including pilgrims, tradesmen) coming from other regions. On the other hand, the reduction of Ne may be explained by the excess of mortality rates and drop of population size in the fourteenth and fifteenth c. due to conflicts [51] as well as multiple outbreaks of plague during the second pandemic as well as other infectious diseases. Notably, the Ne curves for each time group separately showed no evidence of consistent growth or drop dynamics which could reflect heterogeneity in their ancestry and genetic relatedness composition as well as cryptic substructure and other factors that can alter the curve reconstruction. However, plotting the average Ne estimated for each time group over its past four generations seems to provide a reflection of the overall variability of the sample through time. In this way, we were able to overcome the limited signal detection of HapNe-LD by using different time point estimates from the same region.

The overall rate of relatedness in the Sint-Truiden burial ground is lower compared to medieval parish cemeteries in Cambridge in current-day UK [9]. This difference could reflect variations in community size and/or the fact that, before the establishment of the Our Lady (OLV) church in the Sint-Truiden, burials were not organized through a parish. Demographic data is lacking for the earliest periods covered by this study and scarce and problematic for the later centuries. Charles estimates that the town would have had over 3000 inhabitants around the middle of the thirteenth c. and between 4000 and 5000 around the middle of the fourteenth c. ([13], pp. 267–270). Compared to the parish cemeteries of medieval Cambridge, which had a similar size and is the only other medieval town studied genetically extensively so far [9], the individuals buried in the city center of Sint-Truiden had orders of magnitude lower rate of genetic relatedness. This could either reflect a substantially larger local parish population or a wider catchment area for new townsfolk, including the burial of occasional short-term visitors, such as pilgrims.

We did not detect any cases of 1st–7th degree relatedness between individuals (including child–adult pairs) buried together within twelve co-burials examined by us (Additional file 1: Table S11). This suggests that the time of death may have been more important than genetic relatedness in determining co-burials. This pattern was already observed in other medieval cemeteries in Berlin [55] and southern Germany [56]. Additionally, although we found one case of *Borrelia recurrentis* and one case of *Haemophilus influenzae* individually in co-burials (Additional file 1: Tables S11, S16), we found no evidence that the individuals in co-burials had similar metagenomic profiles, indicating that they did not necessarily die of the same infectious disease and were not buried together for that reason. However, these results should be interpreted with caution given potentially high false negative results of these analyses and the fact that RNA viruses and any microbial species with selective tropism would have been undetectable by our screens.

### First direct evidence of fourteenth century plague in Sint-Truiden and the study of its impact on diversity of human immunity genes

Our metagenomic screens revealed in the Sint-Truiden time transect multiple cases of hepatitis B virus (HBV), *B. recurrentis*, roseoloviruses, HSV-1, and *Yersinia pestis*. HBV affects the lives of millions of Europeans today and ancient DNA evidence has shown its presence in Europe since at least the early Holocene [57]. Radiocarbon dates of the ten identified HBV cases in Sint-Truiden cover a broad range from eighth to fifteenth c., suggesting that the virus continued to be a health problem in the city through the Middle Ages. Similarly broad is the time range of the *B. recurrentis* cases, extending from the eighth to thirteenth century. The causative pathogen for louse-borne relapsing fever, which today is only known to cause epidemics in eastern Africa, has been associated with historical record describing relapsing fever epidemics in European history with direct ancient DNA evidence provided so far from Scandinavia and Britain [58, 59]. It is known to have been a major pathogen involved in the health of the European population until the world wars. Being transmitted by human body louse, the findings of this deadly and epidemic pathogen in medieval Sint-Truiden can be indicative of poor hygiene and living conditions in the settlement and represent the first direct evidence of the spread of the disease in the medieval Low Countries.

The validation of ST1516 as an individual carrying *Y. pestis* and the discovery of 5 putative *Y. pestis* cases in individuals dating to the fourteenth c. is historically notable for several reasons. Although the once widely accepted notion that the Black Death bypassed the Southern Low Countries has now been abandoned [60], direct historical evidence of plague mortality in this region during the fourteenth c. is still relatively rare and place-specific [61]. Notably, there are no known references to the presence of the disease in the city of Sint-Truiden and the surrounding Haspengouw area before the fifteenth century. The uninterrupted and otherwise detailed chronicle of the abbey of St Truiden contains later interpolations about the overall European impact of the Black Death, flagellant processions in Germany and Jews being victimized for the pestilence, but says nothing about the plague circulating in Sint-Truiden in this period [15]. Neither do the city’s surviving fourteenth c. administrative sources [16]. Only in the early fifteenth c. do the minutes of the city council (the so-called *maandagboeken*) and other documents mention excess mortality and adversities that can convincingly be linked to the plague [17]. The finding of fourteenth c. *Y. pestis* in Sint-Truiden thus provides another indication that the absence of historical/written records referring to the plague should not automatically be taken as a record of the absence of the disease. It is also remarkable that sequences matching *Y. pestis* could be identified in a fairly high proportion of the individuals from the time group III (1286–1600), almost 10% of the metagenomically screened individuals, which, considering the potentially high false negative detection rate in ancient DNA data suggests that even a larger proportion of later medieval burials in Groenmarkt may represent plague victims. Notably, these individuals were buried in normative single graves, spread out in the cemetery, rather than clustered together in multiple burials. The overall low amounts of *Y. pestis* reads detected for positive individuals despite the high sequencing thresholds of this project, further highlights the difficulty of detection of plague in Sint-Truiden and the likely presence of many false-negative individuals.

Despite the evidence of plague-related mortality in Sint-Truiden in the time group corresponding to the early phase of the second pandemic of plague, we did not observe increased Fst of variants in the innate and adaptive immunity genes when comparing the Sint-Truiden burial cohorts that pre- and post-date the start of the second pandemic. Our results, thus, are not offering support for Klunk et al. [43] observation of significantly higher differentiation of innate immune genes in their London and Danish cohorts dating before and after the Black Death. The Klunk et al. results have been contested on methodological grounds by Barton et al. [44], and similarly to our analyses, the study by Hui et al. [9] was unable to replicate the signal of concerted increase of Fst of many immunity genes across the genome. On the one hand, given the lack of historical evidence of high mortality in Sint-Truiden during the Black Death outbreak, our data set may not be ideally suited for testing such effects. The radiocarbon dates of the Sint-Truiden plague victims are too wide to determine whether any one of them actually died during the Black Death outbreak and our metagenomic data did not allow us to determine which strain the Sint-Truiden plague victims had. However, target enrichment data allowed us to analyze our best samples, ST1516, for the presence of diagnostic SNPs at low coverage. The detection of two post-Black Death variants makes it unlikely the individual died during the Black Death epidemic. The plague victims are therefore likely associated with post-Black Death fourteenth c. epidemics (see SI). Later outbreaks of plague during the second pandemic would have been expected to exert similar selective pressures to the immune genes and yet we do not find evidence of concerted change in the immune genes.

Although we do not detect genome-scale changes in response to the second pandemic in the form of major allele frequency changes in the majority of the immune genes, we cannot rule out the case of natural selection affecting individual immunity genes in relation to the vulnerability to plague and/or other infectious diseases that would have affected the health of the Sint-Truiden population. Our genome-wide screens of Fst outliers (Additional file 1: Table S15) revealed several variants in immune response-related genes among those with the highest allele frequency change in the genome. One of them is the *PLCE1* gene involved in T-cell migration [45], macrophage activation [46], and monocyte counts [47]. Other interesting targets for future validation include *KLHL29*, a gene involved in the lymphocyte count [62].

## Conclusions

The analyses of genetic ancestry changes in the eighth–eighteenth c. burials of the Sint-Truiden city center revealed a genetic homogenization process extending from Early to Late Middle Ages. We found that burials from the same site were structured by ancestry and genetic relatedness both in burial place, by their proximity to the abbey, as well as by time. Over centuries, the Sint-Truiden population became more similar to the current population of the surrounding Limburg province, likely as a result of reduced long-distance migration after the high medieval period, and the continuous process of local admixture of Germanic and Gaulish ancestries, which formed the genetic cline observable today in the Low Countries. We found that individuals carrying genetic variants associated with vitamin D levels are differentiated by their ancestry. Our metagenomic screens detected *Y. pestis* in fourteenth c. context despite the absence of historical records mentioning the presence of plague in the city at that time.

## Methods

### Dating and isotope measurements

Skeletal remains of 83 individuals uncovered from the Sint-Truiden Groenmarkt and Trudoplein excavations in 2018–2020, including 52 whose genomes were studied here, were radiocarbon dated as part of the archeological report of the site [18]. Using these data together with the stratigraphic phase data of the site, four chronological phases of burials could be distinguished: group I predating 1000 AD, group II spanning 1000–1286 AD (up until the construction of the chapel in the Groenmarkt), group III from 1287 to 1599, and group IV from 1600 to 1775. In addition, rib samples of 58 individuals with genomic data were subject to radiocarbon dating. All isotope analyses were performed at the Royal Institute for Cultural Heritage (KIK-IRPA) in Brussels, Belgium. The ^14^C dates were calibrated with OxCal v4.4.4 ([63]; 2021, Bronk Ramsey), using the IntCal calibration curve from [64] and are reported along with isotope values of δ^13^C and δ^15^N for all 110 individuals examined in Additional file 1: Table S1.

### Sampling, decontamination, and extraction

From the skeletal collection of remains, teeth of 404 individuals from Sint-Truiden and 15 from Ypres were sampled for whole-genome shotgun sequencing, including ~ 20% of individuals buried in the Trudoplein around the Sint-Truiden Abbey. Teeth, rather than petrous bone, were sampled to allow the retrieval of both human and pathogen DNA as well as to minimize the invasiveness of the approach. DNA extractions, barcoding, and library preparation for pooled sequencing were carried out in the dedicated ancient DNA laboratory of the Institute of Genomics, University of Tartu. The work largely followed previously described protocols [65]. Per tooth one root was drilled off in a sterile environment. For the decontamination of the samples, surface dirt was removed gently from the root parts using a toothbrush soaked in bleach (6% w/v NaOCl). The samples were then placed in the bleach for 5 min. The roots were rinsed with Millipore ddH_2_O 3 times and were then placed in ethanol for 2 min. The samples were placed on a drying rack under the hood, which was made by placing paper towels on an upside-down multi-rack lid and placing the rack in the drill hood under UV radiation while the roots were getting decontaminated. The UV was left on for more than 2 h to allow the roots to dry. They were weighed while needed reagents (except Proteinase K) and consumables were in the hood under UV. The samples were then transferred to their individual extraction tubes, which were either 5-ml Eppendorf tubes or 15-ml tubes, that contained per 100 mg of each sample 2 ml 0.5 EDTA buffer pH 8.0 and 50 µl Proteinase K. The tubes were placed in a rocker in an incubator at room temperature for 72 h.

Purification of the DNA extracts was achieved by first spinning down the pellets for 5 min at 4000 rpm. After that the supernatants were added to Sartorius concentrators with a 30-kDa filter which were then centrifuged at 4000 rpm for 25–45 min or until extracts were 250 µl. The concentrated extracts were added to 2.5 ml or at least 10X (times the extraction volume) PB buffer inside Roche High Pure Viral Nucleic Acid Large Volume Kit columns which were spun for 1 min at 4000 rpm. One thousand-microliter PE buffer was added to each sample and they were again spun for 1 min at 4000 rpm. The spin columns were removed from the Roche tubes and placed inside clean collection tubes, which were spun at 13,000 rpm for 1 min to dry the membrane. The spin columns were then placed into clean 1.5-ml Eppendorf lo-bind tubes to which 100 µl EB buffer was added. The samples were incubated in a heat block at 37 °C for 10 min and spun at 13,000 rpm for 2 min, after which the silica columns were discarded.

### Blunt-end double-stranded DNA library preparation

Blunt-end double-stranded DNA library preparation was done in 4 steps: End Repair reaction, Adapter Ligase reaction, Adapter Fill-in reaction, and PCR reaction. For the End Repair reaction, 30 µl of purified sample was added to a 0.2-ml PCR tube with End Repair reaction mix consisting of 12.5 µl PCR clean H_2_O, 5 µl End Repair Reaction Buffer (10x) (NEBNext), and 2.5 µl End Repair Enzyme Mix (4 U/μl *T4 DNA Polymerase* and 10 U/μl *T4 Polynucleotide Kinase*) (NEBNext). The samples were then incubated in a Thermocycler for 30 min at 20 °C. To clean the End-Repair reaction mixture, 500 µl PB buffer (Qiagen) was added to each Minelute Spin Column (Qiagen) and samples were spun for 1 min at 13,000 rpm. Then the flow-through was discarded. Next, 690 µl PE buffer was added to each Minelute Spin Column and samples were spun for 1 min at 13,000 rpm. Then the flow-through was discarded, and samples were spun for 1 min at 13,000 rpm to dry the membrane. After that columns were placed in clean Eppendorf low-bind tubes and 30 µl EB buffer (Qiagen) was added to each sample. The samples were incubated for 10 min at 37 °C and spun at 13,000 rpm for 2 min. In the Adaption Ligation step, 30 µl of each end-repaired sample was added to the 20 µl reaction mix, which contained 10 µl Quick Ligation Reaction Buffer (5x) (NEBNext), 5 µl Adaptor mix (2.5 µM) (oligos from Metabion), and 5 µl Quick T4 DNA Ligase (5 U/µl) (NEBNext). The samples were incubated in a Thermocycler for 15 min at 20 °C. After that, the cleaning step described above was repeated. For the Adapter Fill-in reaction, 30 µl of each sample was added to 20 µl reaction mix, which consisted of 12.2 µl PCR clean H_2_O, 5 µl ThermoPol Reaction Buffer (10x) (NEBNext), 0.8 µl of dNTPs (25 mM each) (Thermo), and 2 µl Bst DNA Polymerase, Large Fragment (8 U/μl) (NEBNext). The samples were then incubated in a thermocycler for 30 min at 37 °C followed by 20 min at 80 °C. To prepare the 142 μl PCR reaction mixture, 20 μl of 10 × HGS Reaction buffer (Eurogentec), 20 μl of 25 mM MgCl2 solution (Eurogentec), 10 μl 20 mg/ml Bovine Serum Albumin (BSA) (Thermo), 4 μl 4 × 10 mM dNTPs mixture (Thermo), 4 μl 5 U/μl HGS Taq Diamond Polymerase (Eurogentec), and 84 μl PCR-grade water were used. Four microliters of two different 10 μM indexed primers (NEBNext) were added to each tube. Then, 50 μl of the mixture from the Adapter Fill-in reaction was added to each tube, mixed with a pipette, and 100 μl of each mixture was transferred to another pre-labeled PCR tube. A PCR was run on the samples with conditions: 5 min at 94 °C, followed by 15 cycles of 30, 30, and 30 s at 94, 60, and 68 °C respectively, with a final extension of 7 min at 72 °C and hold at 4 °C. After the PCR was run, the amplified samples were purified using Minelute columns (Qiagen) as it was described before with one change—adding PB buffer repeated twice, both times were added one part of the sample, finally eluted in 35 µl EB buffer.

The DNA concentrations of the libraries were measured with a Qubit® dsDNA HS Assay Kit on a Qubit® Fluorometer. The assay tubes contained 1 µl of sample and 199 µl of Qubit® working solution. Average fragment length per library was determined with Agilent Technologies 2200 TapeStation system or Fragment Analyzer.

### Sequencing

Shotgun sequencing of pooled libraries was performed in two phases: (a) screening phase to determine endogenous DNA content, levels of contamination and sample complexity, and (b) the samples are subjected to further sequencing by mapping of the reads, with a final target of > 0.1 × autosomal sequence depth to allow for sufficiently accurate genotype imputation. The libraries were sequenced at the Genomics Core facilities of KU Leuven using 100 bp paired end read kits on Illumina NovaSeq S4 flow cells for 200 cycles.

### Mapping

All further downstream analyses of human DNA were performed using established pipelines on the cluster of the University of Tartu. Adapters, low-quality 3’ ends, and flanking N bases were removed from the reads using CutAdapt-2.10 [66]. Read pairs were kept if both reads were at least 28 bp long. The paired end reads were merged with FLASH-1.2.11 [67]. The merged reads were mapped to the hs37d5 human reference genome with the BWA-backtrack alignment algorithm of bwa-0.7.17 [68]. BWA-backtrack was chosen because the reads were short length. The resulting files were sorted and converted to BAM files and reads that did not map with the human genome were discarded with SAMTools-1.9 [69]. Duplicate reads were removed with lenient validation stringency using the Picard-2.20.8 tool MarkDuplicates [70]. With the tools RealignerTargetCreator and IndelRealigner of the GenomeAnalysisToolkit-3.5 [71] local realignments around small indels were performed against the reference genome. Known indel site information was provided by an edited version of the dataset Mills_and_1000G_gold_standard.indels.hg19.sites.vcf from the GATK resource bundle. From the resulting BAM files, alignments with a MAPQ (MAPping Quality) lower than 10 were discarded and statistics were produced with SAMTools 1.3.1. A summary of these statistics was reported with a pipeline developed by the Institute of Genomics at the University of Tartu. The proportion of endogenous DNA was calculated by dividing the number of mapped reads including the duplicates with the total number of reads.

### Quality and ancient DNA authenticity tests

Damage was measured by calculating the rate of C to T transitions in the ends of 5′ to 3′ reads with mapDamage2.0.6 [72]. Contamination rates were estimated for autosomal DNA with ANGSD-0.917 [73], which has a contamination analysis module for the X-chromosome based on a maximum likelihood method as described in [74]. It puts out a moment-based estimate of the mismatch rate and a Bayesian-based estimate of the posterior probability of the contamination rate. Polymorphic sites were identified in the HapMap CEPH [75] individuals and the error rate at the sites was compared to the error rates at adjacent sites. Two test methods were used, test 1 considers the mismatch rates both within and between SNPs as independent, while test 2 uses only one randomly sampled read. For mitochondrial DNA, the rates of contamination were estimated by calculating the percentage of non-consensus bases at haplogroup-defining positions as described in [76]. All samples were mapped against the hs37d5 human reference genome and checked against haplogroup-defining sites for the sample-specific haplogroup.

### Sex estimation

After filtering for alignments with MAPQ > 20 with SAMTools-1.9, the sex of the samples was inferred with two different methods: ry_compute [77] and karyo_RxRy [78] (Additional file 1: Table S1). The methods do this by calculating the ratio of reads mapping to the X and Y chromosomes. They were run with default settings according to the documentation.

### mtDNA haplogroup determination

Mitochondrial variants were called for all individuals with bcftools version 1.14 [79] by first calculating genotype likelihoods with the mpileup function using the mitochondrial hs37d5 sequence from 1000 genomes phase 2 [80] as a reference and then calling the variants with the –multiallelic-caller option. Mitochondrial haplogroups were determined with HaploGrep version 2.1.21 [81] which used the mtDNA tree built by PhyloTree Build 17 [82].

### Y chromosome variants calling and haplotyping

Binary Y-chromosome variants were called at ~ 273,000 sites that have been detected as polymorphic in previous high-coverage whole Y chromosome sequencing studies [83–85] with ANGSD-0.917 using haploid calling and no reference. The resulting files were converted to plink format with the haploToPlink tool of ANGSD. Those files were then converted to vcf and bed format with PLINK-1.9.0. The SNP positions were compared with reference and alternative alleles and were removed if an allele other than those was observed. The diploid output was converted into haploid format and each SNP was annotated with bedtools-2.29.2 [86]. Sub-haplogroup assignments, as reported in Additional file 1: Table S1, were determined on the basis of mapping the derived allele calls to the internal branches of the YFull tree [87], requiring the support of at least two variants for the terminal branch assignment.

### Pseudohaploid genotyping

For the genetic relatedness analyses, SNPs were haploid called at 4,359.855 biallelic single-nucleotide variant sites within the UK10K subset of the HRC panel [88] filtered for a MAF (minor allele frequency) of more than 5% from 382 BAM files of 382 Sint-Truiden individuals with coverage above 0.01. One randomly sampled allele was called with ANGSD-0.917 using the –doHaploCall 1 option, without reference, at 5.5 million common variants in modern reference data. The UK10K haplotype reference panel [89] was used as the reference for common variant defining as geographically and genetically the closest proxy to the Belgian population in the HRC panel. The output was converted to a PLINK tped file using ANGSD. With PLINK-1.9.0 [90], the tped file was converted to vcf and bed format.

### Screens of 1st–3rd degree relatedness

To screen for the presence of closely related (1st to 3rd degree) individuals, KIN [34] and READ2 [35] were applied on 372 individuals with coverage > 0.01x. We found that among the 70,790 individual pairs with more than 30,000 overlapping SNPs according to READ2 the exact same 1st to 3rd degrees of relatedness classifications ascertained by KIN were also found with READ2.

### Imputation

The genotypes of all Sint-Truiden and Ypres individuals were imputed with QUILT 1.0.2 [91] using a reference panel with haplotypes from the HRC version 1.1 [88] with variants with a MAC (minor allele count) below 5 filtered out. To optimize computation efficiency, the genome was divided into 771 overlapping segments. The 771 segments were concatenated with bcftools v. 1.14 after imputation. BCFtools command tag2tag (–gp-to-gt option) was used to correct conflicting genotypes (GT field) by respective GP field values. All imputed batch files were merged together using BCFtools merge command, resulting in a file with 40.5 million variants. Variant positions with minor allele frequency less than 5% (MAF < 0.05) in the HRC reference panel were removed as their imputation accuracy is expected to be low [91, 92], keeping 6.1 million variants. Duplicate variants were removed, and with PLINK 1.9 individuals with a coverage below 0.1 were removed, leaving 338 Sint-Truiden individuals and 8 Ypres individuals. We then merged the resulting bed files with 288 genomes from the Sasso et al. data, including data from Gretzinger et al.. In downstream analyses, we included 4.7 million SNPs with less than 1% missing rate.

### Principal components analysis

The resulting 4.7 million Sint-Truiden variants were merged in PLINK with a dataset containing other Early Medieval [5, 6], Iron Age French Gaul [22], and modern European reference genomes from the UK Biobank [32] and MinE consortium data [26, 27]. The merged data set included 238,449 variants in total. For the PCA, the merged data were pruned for linkage disequilibrium using PLINK 1.9 with a window size of 1000 variants, a step size of 50 variants and a pairwise r^2^ threshold of 0.5. The likely non-neutral regions exclusion_regions_hg19.txt were also filtered out. The first four principal components were then calculated with FlashPCA 2.0 [93] without using projection.

### Population structure analyses

#### Dataset

The population structure analyses are based on the initial dataset of imputed genotype data downsampled to 600 K SNPs available in the Allen Ancient DNA Resource dataset (AADR) [94, 95]. The analyses include 332 Sint-Truiden (ST) individuals (ID label: ST*N*, Additional file 1: Table S1) and additional medieval, Roman Period and Late Iron Age reference data [5, 6, 8, 22–25] from Belgium, the Netherlands, the UK, Denmark, France, Germany, and Viking Age Orkney, as well as modern reference data from Belgium (BE, the MinE consortium data [26, 27]), the Netherlands (NL, MinE consortium data), data from the UK and Denmark (the UK Biobank, [32]), along with Northern Italian [96] and YRI (Yoruba in Ibadan, Nigeria) groups [80].

#### Outliers

Based on previous studies, we removed from all population structure analyses: sample 3DT26, a Medieval York sample of Syrian ancestry [25], three samples from the French Gaulish group (UN19, UN85, UN129, [22])and one sample from the Ireland Celtic group (KIL042) [5].

#### Supervised ADMIXTURE

Supervised ADMIXTURE [97] was performed by modeling the target groups as a two-way admixture. As reference sources, we used Late Iron Age (LIA) genomes (*N* = 15) from France [22] as a proxy for Gaulish Celtic ancestry and early medieval (EMA) genomes (*N* = 22) from the Netherlands [5] as a proxy for Germanic ancestry. The analysis was carried out on both modern and ancient samples, pruning the two groups separately (PLINK 1.9 –indep-pairwise 50 10 0.1), eventually retrieving over 100 K SNPs, as per manual recommendation [90, 97].

We used the option –supervised and -B 200 to perform supervised ADMIXTURE with bootstrapping and report the standard errors along with the ancestry assignments.

#### qpWave & qpAdm

qpWave and qpAdm were performed with the software AdmixTools [98], using the following three right sets, consisting of populations with “differential relatedness” with the proxy sources [99]: (1) R1: YRI (*N* = 108), North Italian (*N* = 21) [96, 100], Germany North Rhine EMA (*N* = 7) (5), Cambridge EMA (*N* = 46) (5), Denmark EMA (*N* = 7) (5), Germany Low Saxony EMA (*N* = 26) (5), Germany Corded Ware (*N* = 16) [101]; (2) R2: Han (*N* = 45) [96, 100, 102], North Italian, Germany North Rhine EMA, Cambridge EMA, Denmark MA, Germany Low Saxony EMA, Germany Corded Ware; (3) R3: North Italian, Germany North Rhine EMA, Cambridge EMA, Denmark EMA, Germany Low Saxony EMA, Germany Corded Ware. All three right sets produced qpWave models with *p*-value < 0.01; however, only R3 produced a significant model with qpAdm *p*-value > 0.05 and was used in all further downstream analyses. We used inbreed: YES in qpAdm runs where the target sample size was > 1, and inbreed: NO where the sample size equals to 1. In all analyses, we used allsnps: YES.

#### HapNe-LD

We used the software HapNe-LD [40] to estimate the genetic effective population size (Ne) of each time group over its past 4 generations. Closely related individuals (1st to 3rd degree) were identified by running READv2 [35] on each time group independently and a member of every pair was discarded. We favored the removal of individuals involved in multiple pairs of genetic relatedness in order to maximize final sample sizes, to which HapNe-LD is particularly sensitive [40]. Config file settings were left as default. The evolution of Ne over time was eventually obtained by plotting the arithmetic mean of Ne computed for each time group over its past 4 generations. The 95% confidence interval was determined following the same approach.

### Detecting IBD segments

IBD segments and kinship coefficients were estimated from the merged PLINK files of 346 imputed ancient genomes from Sint-Truiden and Ypres (this study), Early medieval (EMA) genomes from Gretzinger et al. [5], Sasso et al. [6], Late Iron Age (LIA) genomes from Fischer et al. [22], Viking Age (VA) data from Margaryan et al. [8], Roman period data from Scheib et al. [23], Martiniano et al. [25], and Schiffels et al. [24], a subset of modern European reference data from the 1000 Genome Project [80], MinE [26, 27], and UK Biobank data with IBIS 1.20.9 [33] using thresholds of minimum shared segment length (-min_L) of 5 cM and/or 7 cM (in genetic relatedness analysis, Fig. 6B, Additional file 1: Table S9) together with parameters -a 0, a minimum kinship coefficient of 0.0001 and -maxDist 0.1. In total, 203,995 binary SNVs with MAF > 0.05 were used.

In genetic ancestry analyses (Fig. 5A), we used > 5 cM threshold in our diachronic inferences of population affinities because our focus is on relationships at generational distances > 15 at which longer IBD sharing expectations become relatively low [103], especially combined with the loss of sensitivity to detect long IBD segments from imputed ancient DNA sequences.

The probability of individual connectedness (PiC) score for individual x in group Z was estimated as the proportion of individuals from group Z with whom individual x shared IBD above the given threshold. We estimated the count of connected individuals from group Z from sorted IBIS.coef output files by using the linux “join” function to add group codes to individual identifiers and by using the “crosstab” function of datamash [104] to generate the table of counts, each of which we divided by the total number of individuals in group Z to obtain individual connectedness proportions by groups (the PiC scores).

### Runs of homozygosity

We used hapROH [36] to detect runs of homozygosity (ROH) in 332 imputed ancient genomes with coverage > 0.1 ×. This analysis was performed using both the pseudo-haploid and diploid emission models with default parameters. Among the imputed common variants in the HRC panel, 849,620 variants overlapped with the 1240 K SNP panel used in hapROH. To assess consistency, we compared the inferred ROH tracks with ROH calculated from non-imputed pseudo-haploidized data, commonly used for this analysis. We used the program pileupCaller from the sequenceTools package (v1.5.3.2) [105] to genotype the Sint-Truiden genomes selected for imputation. A pileup file for the 1240 K SNP sites was generated using samtools mpileup with parameters -q 30 -Q 30 -B. From this pileup file, a single read covering each SNP was randomly selected, and the individual was assumed homozygous for the allele on the selected read for each SNP (i.e., pseudo-haploidized). Finally, 255 Sint-Truiden genomes with at least 400,000 of the 1240 K sites covered were retained for analysis in hapROH using the pseudo-haploid emission model with default parameters. Genome-wide heterozygosity was estimated with the –het function in PLINK 1.9 for the 332 imputed Sint-Truiden genomes.

### Enrichment of immune genes

To test the enrichment for higher allele frequency differences at variant positions of immune genes in cohorts pre- and post-dating the start of the 2nd pandemic of plague, we used PLINK –fst case–control as in Hui et al. 2024. This was performed on a list of variants with minor allele frequency higher than 10% in imputed Sint-Truiden genomes, excluding the five outliers identified by PCA. The examined shortlist of variants included 50,505 variants from the 37,574 putatively neutral regions defined by Gronau et al. [106], a list of 16,823 variants from 189,172 exonic regions of the 4723 innate immunity genes curated by InnateDB [107] and 2636 variants from 4 adaptive immunity genes from VDJbase [108] and 129 exonic regions of the HLA complex. The Sint-Truiden genomes were divided between two cohorts, dating to before (time groups I, I/II, and II: *n* = 229) and mostly after (time groups III/IV and IV: *n* = 46) the start of the second pandemic of plague. Variants that had an Fst in the immune genes above the neutral 99th percentile threshold in the putatively neutral regions were considered as highly differentiated (Additional file 1: Table S14). We used PLINK –indep-pairwise 1000 50 0.1 function to determine the numbers of independent variants (Additional file 1: Table S10) and binomial probabilities to explore the enrichment of the detected signals.

### Genome-wide scans of high Fst variants

To investigate possible selection signals at the level of individual variants, we screened the entire genome for variants with high Fst between time groups. Firstly, we removed variants in linkage disequilibrium (r2 > 0.1) with PLINK –indep-pairwise function and variants with minor allele frequency (MAF) < 0.1, retaining in total 37,403 variants. Then, we estimated for each variant the Fst value using PLINK v2 [90]. We were interested in the increased level of differentiation between the earlier time groups (group II) and the more recent time groups (III and IV). In order to remove the effect of random drift, we collected the values that fall over the 95th percentile when comparing group II versus group III, group II versus group III/IV, and group II versus group IV, then we selected only the SNPs that fall over the 95th percentile of the genome-wide distribution in all comparisons. All the variants were annotated using VEP [109].

### Analyses of variants with strong phenotypic effect

To filter out the bad variants from the imputed VCFs, we used the dosage (DS) parameter, keeping the variants with DS = ± 0.1 from the expected values (i.e., 0 for homozygous genotype for the reference allele, 1 for heterozygous genotype, 2 for homozygous genotype for the alternate allele). From the imputed samples (discarding those with an initial coverage < 0.1 x), we then extracted the genotype information at 112 SNPs [110, 111] involved in diet, disease, and pigmentation phenotypes. More specifically, we analyzed 24 SNPs involved in diet adaptation and metabolism, 49 in immunity response, and 39 from the HIrisPlex-S [112] set for the prediction of eye, hair, and skin colors (Additional file 1: Table S12). For the pigmentation prediction, we prepared an input file for the HIrisPlex-S webtool (https://hirisplex.erasmusmc.nl/) following its manual for formatting and results interpretation. Sample-by-sample phenotype prediction and genotypes (as counts of the effective alleles in the form 0, 1, or 2) are reported in Additional file 1: Table S13, while sample-by-sample genotypes of the other markers are reported in Additional file 1: Table S19. Variants with strong predictive effect on red hair used in the analyses allele frequencies between two ancestry groups were as follows: rs11547464_A, rs1805008_T, rs1805006_A, rs1805007_T, and rs1805009_C.

We then grouped the ancient Sint-Truiden individuals in two different cohorts: individuals belonging to time group I and/or II and individuals which belong to time groups III and/or IV and estimated the allele frequency of the phenotypic markers. We then compared the allele frequencies of the 112 SNPs in the genomes of the time group I/II individuals with reference data of 112 modern Flemish genomes from the MinE dataset [26, 27] performing an ANOVA test and applying Bonferroni’s correction on an alpha value of 0.05 for the number of tested SNPs to set the significance threshold.

### Metagenomic analyses

Raw sequencing data were merged by sample, and data quality was assessed with fastqc [113]. Data was then trimmed and quality filtered with cutadapt [66], which was run in paired-end mode with the pair-filter on “any” (-m 30 –nextseq-trim = 20 –times 3 -e 0.2 -j 0 –trim-n). Reads were subsequently merged with Flash2.0 [67] (-z -M 125). Final dataset quality was determined with multiqc [114] and analyzed with KrakenUniq [115] to detect the presence of human associated pathogens and pathobionts. The database used for analysis included dusted complete genomes and chromosome-level assemblies of bacteria, viruses, archaea, and protozoa, the human genome, the NCBI Viral Neighbor database, and the contaminant databases UniVec and EmVec. For visualization and evaluation of the results, heatmaps and tables were generated in python using plotly [116], pandas, matplotlib [117], and numpy [118]. E-values were calculated as described in Guellil et al. [119]. E-value cut-off for further inspection was 0.006 due to the high sequencing depth represented in these samples. Reported taxa were further validated by mapping the data to matching reference sequences using bwa aln (-n 0.04 -l 1000) [68] with samse. Resulting SAM files were converted to BAM format, sorted and filtered for mapped reads with samtools [69]. Picard’s [70] MarkDuplicates module was used to remove duplicates from the mapping. Misincorporation patterns were computed using mapDamage (v2.2.1) [72]. Mapping plots were visualized using aDNA-BAMPlotter [120] and mapping statistics were calculated with Qualimap [121] and using python modules pysam [122], pandas, biopython [123], and numpy [118].

Sample from individual ST1516 (OLV255 in Additional file 2: Fig. S12) was our best hit for the plague pathogen *Y. pestis* and was subsequently enriched using a custom *Y. pestis*/*Yersinia pseudotuberculosis* myBaits target enrichment kit from Daicel Arbor Biosciences (v4) [124]. The design encompasses the *Y. pestis* CO92 reference sequence (including all plasmids) and the *Y. pseudotuberculosis* reference sequence (NC_006155.1). The sample was enriched following the myBaits v5 protocol with the exception that only half a bait aliquot was used. Amplification of the final capture product was performed using 2X KAPA HiFi HotStart ReadyMix DNA Polymerase and primers IS5 and IS6 [125]. The enriched library was then sequenced on a NextSeq500 (MID150, PE) at the Estonian Biocenter Core Facilities at the University of Tartu. Samples from individuals ST851, ST657, ST1358, ST1484, ST1516, ST815, and ST1319 were also enriched with less success (see SI).

## Supplementary Information

Additional file 1: Supplementary tables.

Additional file 2: Supplementary information and figures.

## Data Availability

The sequencing data supporting the conclusions of this article are available in the European Nucleotide Archive (ENA) repository at EMBL-EBI, under Accession No. PRJEB79468. Isotope analysis and radiocarbon data are available in the Figshare repository, under 10.6084/m9.figshare.28731323. External sequencing datasets used for our analyses are those part of the Allen Ancient DNA Resource dataset (AADR) [4, 5, 8, 24, 25, 31, 39, 76, 80, 96, 98, 100–102, 110, 111, 126–325] as well as several other datasets [6, 22, 23, 26, 27, 30, 32, 242, 326].
