# Supplementary material for: Urbanization and genetic homogenization in the medieval Low Countries revealed through a ten-century paleogenomic study of the city of Sint-Truiden
Source: Genome Biol. 2025 May 20;26:127. doi: 10.1186/s13059-025-03580-z (PMC12090598; doi:10.1186/s13059-025-03580-z)
Supplement: Supplementary file 2 — Additional file 2: Supplementary information and figures. [file 13059_2025_3580_MOESM2_ESM.docx]

**Urbanization and genetic homogenization in the medieval Low Countries revealed through a ten-century paleogenomic study of the city of Sint-Truiden**

Supplementary information

**Archeological background of the studied sites**

Description of the city center of Sint-Truiden site, Belgium

From 2018 to 2020, Aron bv excavated [(De Winter, 2023)](https://www.zotero.org/google-docs/?ZQUelz) in the center of the city of Sint-Truiden, for the redevelopment of the Trudoplein square, the Groenmarkt city square and surrounding streets. The Trudoplein borders the tower of the abbey church of the former St. Trudo Abbey to the south (fig. 1). The Groenmarkt, in turn, borders the Trudoplein to the south. To the east of this market square is the parish church, the Church of Our Lady (OLV Church). In the south the market is bordered by the town hall. The excavations at the Groenmarkt site were executed in phases and this site can consequently be subdivided into four great zones.

The history of the city of Sint-Truiden has always been closely linked to the Sint-Trudo Abbey. This settlement gradually grew around the church and monastery that were founded by Trudo, a nobleman of Frankish descent, in the second half of the 7^th^ c. on the family domain of *Sarchinium*. The existence of such a settlement was already mentioned in the second half of the 8^th^ c. by *Donatus* in his *Vita Sancti Trudonis*.

The excavated area was used for burials for centuries. The vast majority of the archeological traces found consisted of graves. A total of 3046 different individuals could be distinguished, 123 from the Trudoplein, the rest from Groenmarkt, the oldest of which date from the end of the 7^th^ c. or the 8^th^ century. In the early Middle Ages, the cemetery extended over a large area: from the abbey tower to the current town hall. Even after 1000 AD, burials continued around the abbey tower on the current Trudoplein. The youngest grave from the area near the tower is from the 14^th^ c., after which burials stopped at this location. People also continued to be buried on the Groenmarkt after 1000, but the cemetery there now moved gradually in an easterly direction. In the second half of the 11^th^ c., the Church of Our Lady was built east of the Groenmarkt, by 1286 when the Clerics Chapel was completed the cemetery wall was already established in the south. From the end of the 13^th^ c., possibly a little earlier, burials were carried out within the cemetery walls, in zones 1 and 2 only (Figure 1). The western part of the Groenmarkt, with burial zones 3 and 4 in the preceding period, must have been used as a market from then on. The eastern part included a part of the cemetery around the Church of Our Lady, which was finally abandoned in the second half of the 18^th^ century.

The deceased from Sint-Truiden were usually buried in separate pits and coffins, lying on the back, the head approximately in the west, the face directed towards the east. In some cases, however, “different” burial methods were identified. 208 individuals were buried in 80 “multiple graves”, in which more than one person lay buried in one grave. The most common form of multiple graves was one with an adult person (20 years or older) buried with one or two sub-adults. It seems that the deceased had died a natural death since no traces of violence could be determined as a cause of death. 31 other individuals found were buried in anthropomorphic coffins, coffins which follow the shape of the body. Furthermore, one person was found buried in a hollowed-out tree trunk and another one in a brick vault. All these deviating ways of burial seem to be limited to the oldest graves in Sint-Truiden. The deviant graves that were dated, strictly belonged to periods predating the mid-12^th^ century. Afterwards there seems to be a more standardized way of burial.

Description of the Hooge Siecken leprosarium site at Ypres, Belgium

In 2018, infrastructural works in preparation of a new housing estate led to the discovery of the preserved remains of the leprosarium (and later farmstead) site of which the exact location was previously uncertain and the state of preservation unknown [(Vanhoutte *et al.*, 2022)](https://www.zotero.org/google-docs/?PnCHZ8).

Like many medieval towns, the city of Ypres opened a leprosarium already in the 12^th^ c., probably before 1168, just outside the city gates. Soon after, the population of Ypres expanded so considerably that by the early 13^th^ c. the leprosarium had to make room for the development of an outer parish. A new leprosarium was built at a 1 km distance from the city gates, at Sint-Jan, on higher grounds and strategically positioned along the main road coming from Bruges. The name of this leprosarium has been preserved as *Hooge Siecken*. Based on the archival documents, its founding date can be set at *c.* 1230, certainly before 1236. It is this leprosarium that has yielded the 15 skeletons that are also analyzed in this project.

In the 14^th^ c. the leprosarium of the *Hooge Siecken* became one of the three principal leprosaria in the County of Flanders, next to Ghent and Bruges. These three research centers had the monopoly of the research right, the visitation or inspection of leprous sufferers [(Maréchal, 1976)](https://www.zotero.org/google-docs/?pEJ9Bj). Ypres was the center for leper inspection for the castellanies of Ypres (Ieper), Courtrai (Kortrijk) and Oudenarde (Oudenaarde), and was even consulted by leprous sufferers from Dendermonde. For the period of the peak of leprosy, Viaene 1962 assumes that 80 to 100 new cases a year were inspected at the leprosarium of the *Hooge Siecken*. Charters give evidence for 3184 candidates from these areas for a visitation at *Hooge Siecken* in the period 1549-1583 [(Mus, 1950)](https://www.zotero.org/google-docs/?T8cDmk). It was a civic leprosarium reserved for burgesses [(Viaene, 1962)](https://www.zotero.org/google-docs/?sbaY8A). The fifteen samples from this site are presumed to be representative for this region in the High/Late Middle Ages. A first selection of ten individuals has yielded a radiocarbon dating in the late 13^th^ and 14^th^ centuries for this fringe of the cemetery.

**Capture analyses of samples identified with *Yersinia pestis* reads.**

During our screening we identified seven samples (ST851, ST657, ST1358, ST1484, ST1516, ST815 and ST1319) with reads matching the causative pathogen for plague, *Yersinia pestis*. The samples were enriched using a custom capture panel (see methods). Initial low detection of *Y. pestis* reads was possible due to high sequencing thresholds during shotgun sequencing, but DNA preservation was limited and enrichment was needed to verify the identification. However, based on the result of the enrichment, ST1358 could not be confirmed as an *Y. pestis* infection and the data for ST851 remains too limited to validate the sample. Coverage for ST815, while also very limited, was spread across all specific plasmids, allowing for a validation of the *Y. pestis* infection. ST1516, ST657, SK1484 and SK1319 could be validated.

Of all samples for which we identified reads for *Y. pestis*, SK1516 had the best coverage following capture. The sample was able to reach 0.153X mean depth of coverage with 7% of the chromosome being covered at least once and reached between 7-49% (1X) sequence coverage across the *Y. pestis* plasmids (using both shotgun and capture data). To determine whether the plague victims identified in Sint-Truiden died during the Black Death epidemic or in following epidemic events of the Second Plague Pandemic, we mapped reads to the *Y. pestis* reference genome CO92 and called SNPs using bcftools (1.6) [(Li *et al.* 2009 )](https://paperpile.com/c/oY5Gxc/7rxO) and freebayes (1.3.5) [(Garrison and Marth, 2012)](https://www.zotero.org/google-docs/?gUKUuj). However, as the mean depth of coverage is under 1X we called alternative alleles with min 1X, MQ>30 and >90% alternative alleles. This allowed us to analyze the sample for the presence of diagnostic mutation accumulated during the Second Plague Pandemic following the Black Death. Of the 125 positions listed in Keller *et al.* 2023 [(Keller *et al.* 2023)](https://paperpile.com/c/oY5Gxc/OEHM), eight were covered by at least one read in OLV255. Of these, six were reference calls and two SNPs. The two SNPs, pos. 1,952,848 (G>A) and pos. 3,872,698 (C>T), are not present in Black Death strains but present in all strains in the main Second Plague Pandemic lineage (and not present in any of the *pestis secunda* strains), starting with the basal branch 1A. Indeed, based on the dating of the sample and the presence of these SNPs, we hypothesize that the outbreak that likely killed the plague victims identified in Sint-Truiden was one of, or multiple of, the early waves of plague, which hit the region following the arrival of plague in Europe during the Black Death, as exemplified by the strain AHM011 (Arnhem, Netherlands) [(Keller *et al.* 2023)](https://paperpile.com/c/oY5Gxc/OEHM). It should be noted that the coverage of this sample was low and so were the call filtering criteria. Additionally, deamination could be an issue. The middle of the read position of the mismatch covering 3,872,698 (C>T) (pos. 46 from 5' of read; fragment length is 68bp) and the fact that most damage is observed in the read ends makes it less likely that the observed variant allele is caused by deamination. In case of 1,952,848 (G>A), the likelihood of deamination is higher, as the base covering the SNP are situated at the 3' end of the read (pos. 6 on read from 3' end of read; fragment length is 78bp), where based on mapDamage2 ca. 3.4% (read pos 6 - 3' end) of reads are still expected to carry G>A misincorporations in our mapping. However, considering the co-presence of these two mutations in many second pandemic samples, low number of SNPs called for SK1516 and the low mutation rate of the bacterium in general, the combined presence of these calls is more likely to reflect real mutations than damage.

Supplementary figures

**
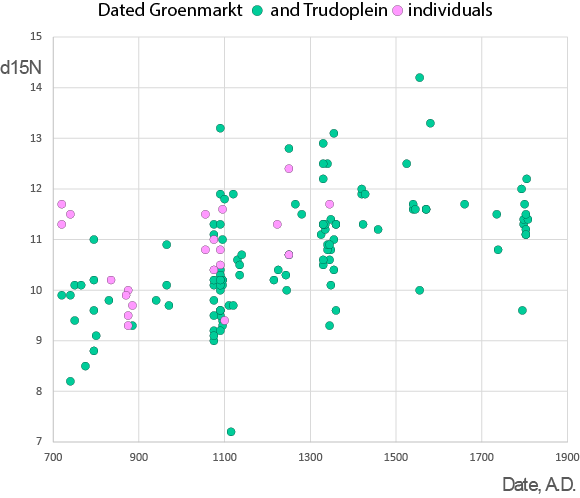
**

**Fig S1 Relationship between radiocarbon dating and δ15N values as indicators of diet in Sint-Truiden burials.**


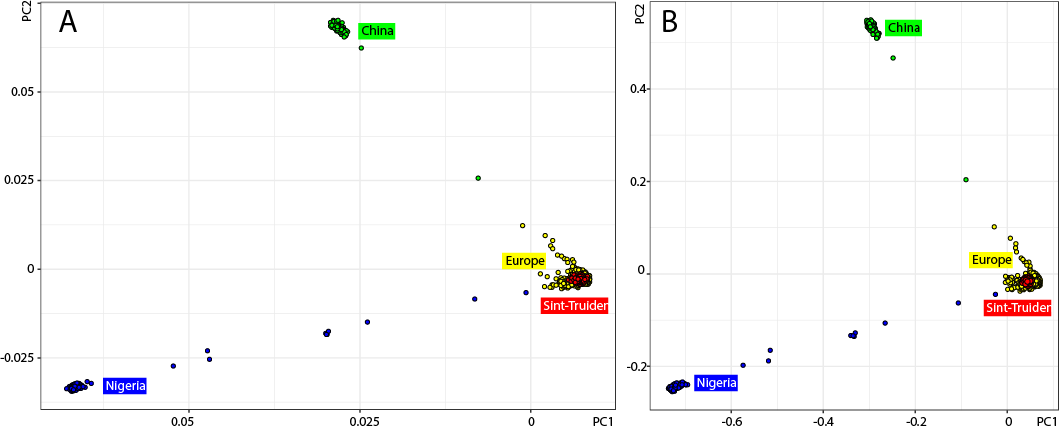


**Fig S2 Principal component analysis of Sint-Truiden genomes in the global context.** A. ancient Sint-Truiden genomes projected on PCA generated from modern genomes with eigensoft/smartpca; B - PCA generated with flashPCA2 from ancient and modern genomes without projection. Ancient genomes from Sint-Truiden – 329 imputed genomes, this study; Modern genomes: China – 192 individuals with birthplace in China from the UK Biobank (Bycroft *et al.*); Nigeria – 193 individuals with birthplace in Nigeria from the UK Biobank, Europe – 2068 individuals with birthplace in Spain, France, the Netherlands, the UK, Ireland and Scandinavia from the UK Biobank.


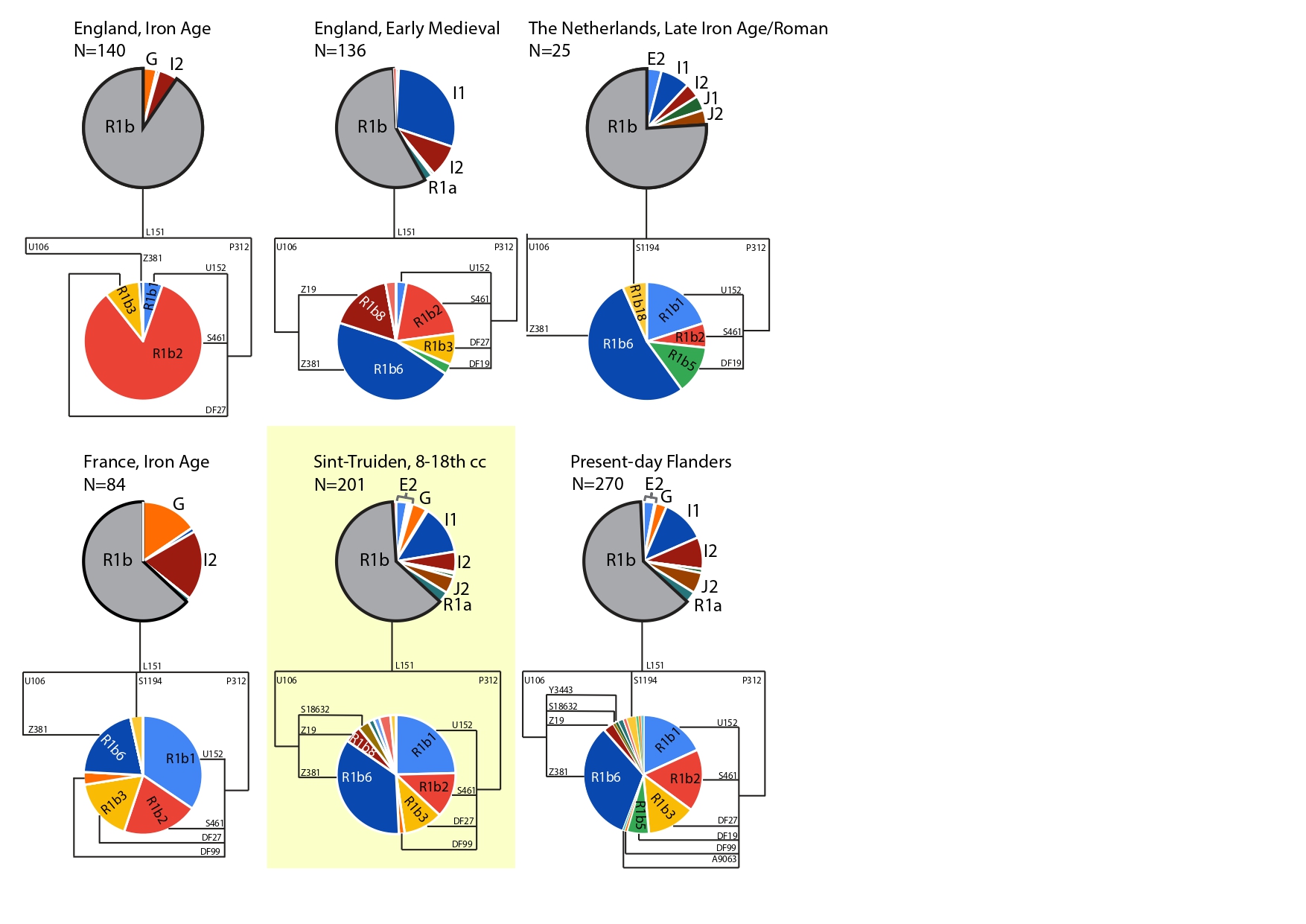


**Fig S3 Y chromosome haplogroup frequencies in 8–18th century Sint-Truiden in regional and temporal context.** Sources: Sint-Truiden (at light yellow background) - this study, present-day Flanders (Larmuseau *et al.*), Late Iron Age France (Fischer *et al.*, McColl *et al.*), Late Iron Age/Roman Netherlands (McColl *et al.*), Iron Age England (Patterson *et al.*), Early Medieval England (Gretzinger *et al.*). Further details are provided in Additional file 1: Tables S1 and S7. Haplogroup names are shown according to Karmin *et al.* 2015 nomenclature. Haplogroup R1b main sub-clade defining marker names are shown above each relevant branch.


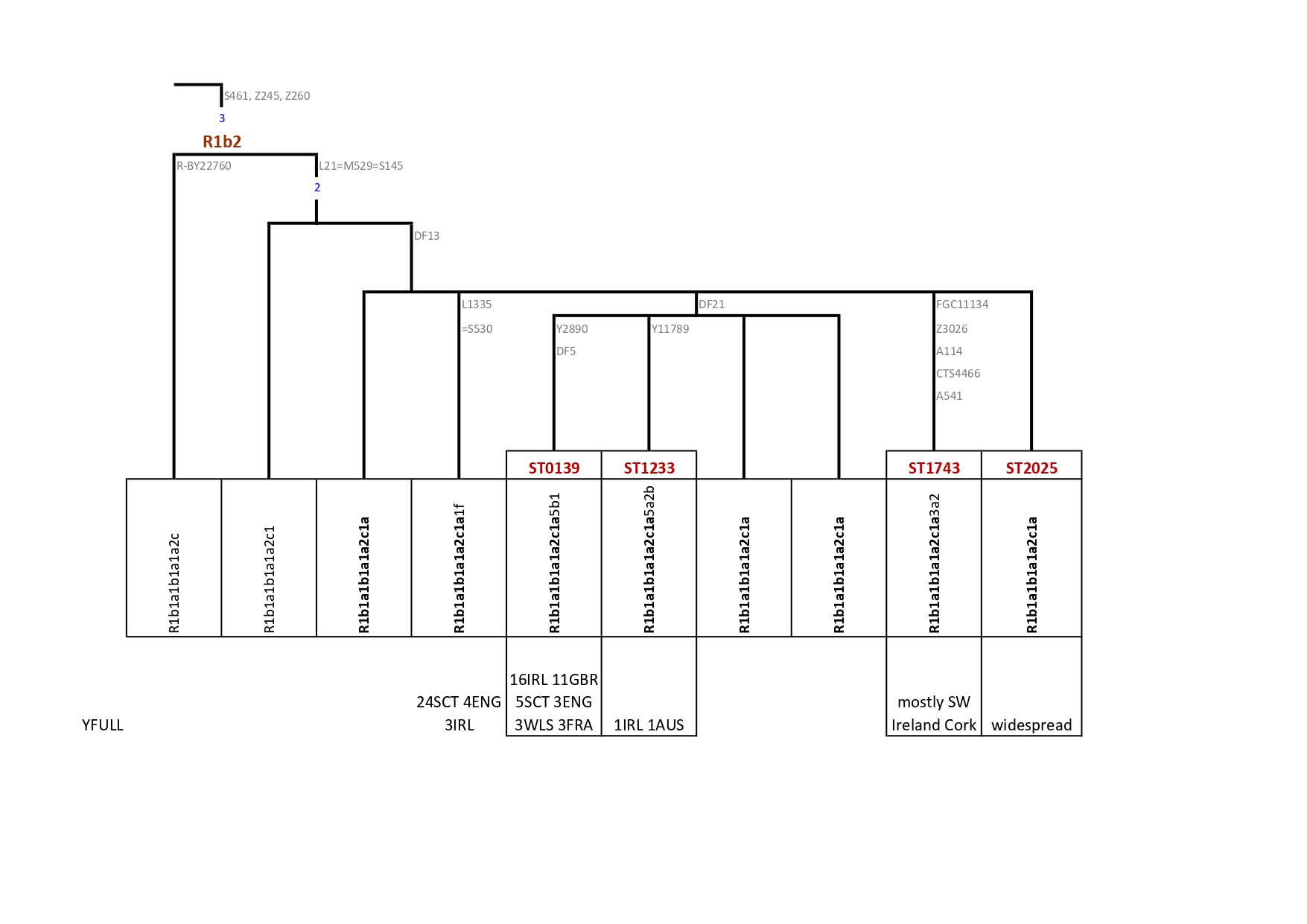


**Fig S4 Phylogeny of Y chromosome haplogroup R1b2 subclades with four Early/High Medieval individuals from Sint-Truiden identified as PCA outliers with autosomal ancestry from Scotland or Ireland.** Numbers of individuals by country within the YFull data (https://www.yfull.com/) are shown below the phylogeny. IRL – Ireland, ENG – England, SCT – Scotland, WLS – Wales, GBR – Great Britain, AUS – Australia, FRA – France.


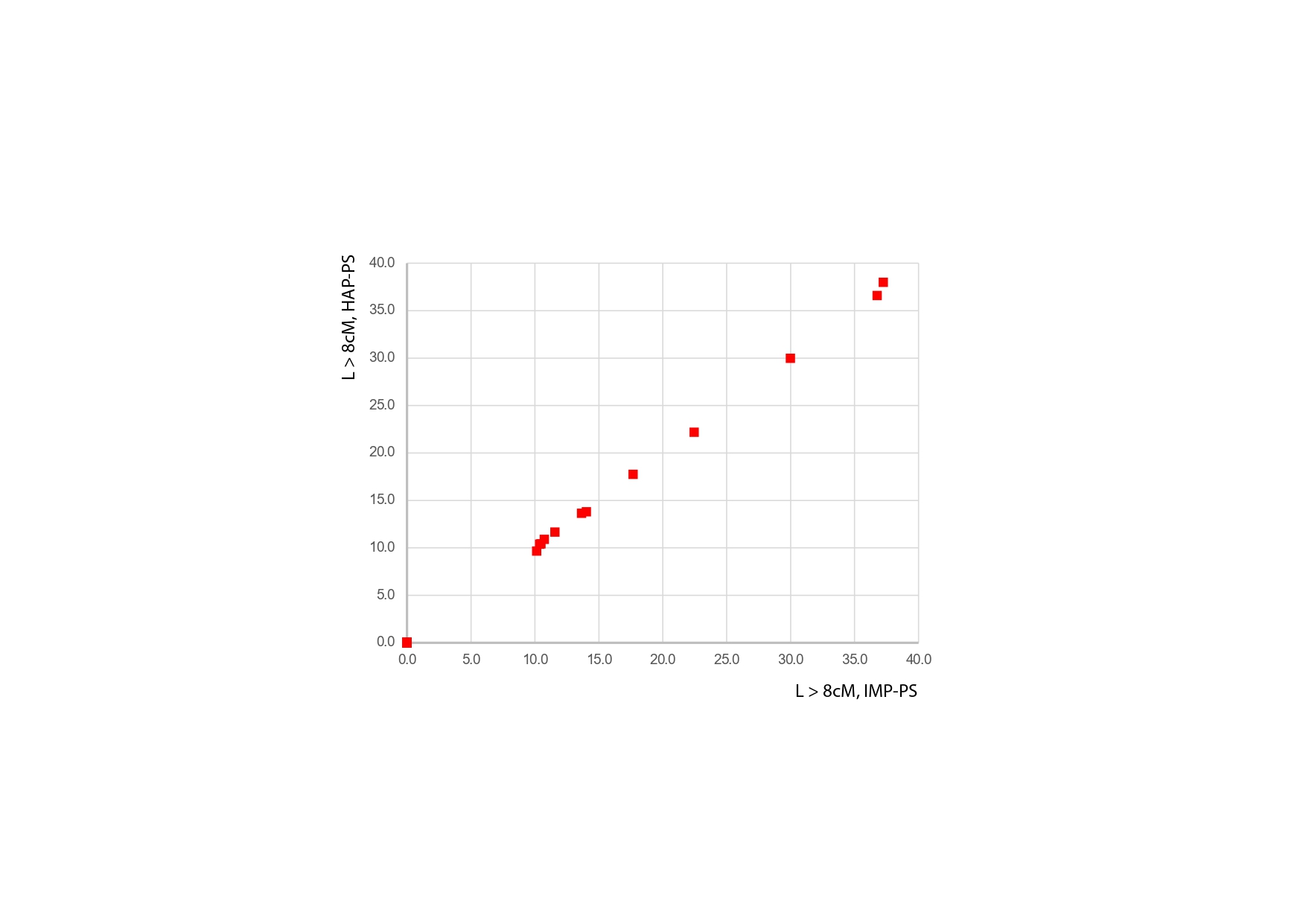


**Fig S5 Runs of homozygosity >8 cM detected in Sint-Truiden genomes.** The scatter plot shows total lengths (L) of runs of homozygosity detected with hapROH (Ringbauer *et al.*) with pseudo-haploid (PS) model using imputed (x axis, IMP) and haploid-called (y axis, HAP) data as input.


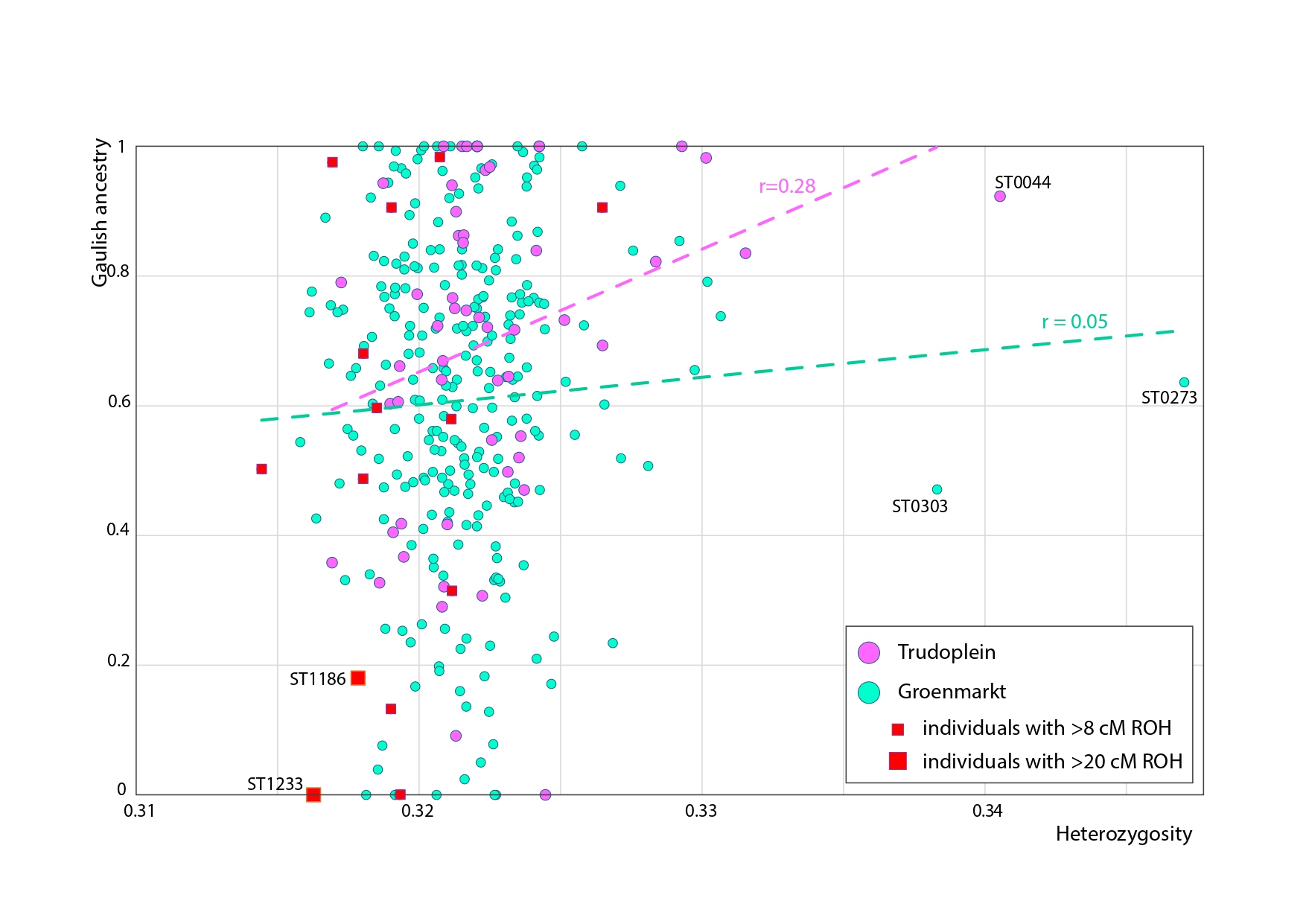


**Fig S6 Heterozygosity and Gaulish ancestry in Groenmarkt and Trudoplein burials.** Individuals with >8 cM ROH segments are highlighted in red (they all come from Groenmarkt). The IDs of three outliers with high heterozygosity at MAF >0.05 sites are shown.


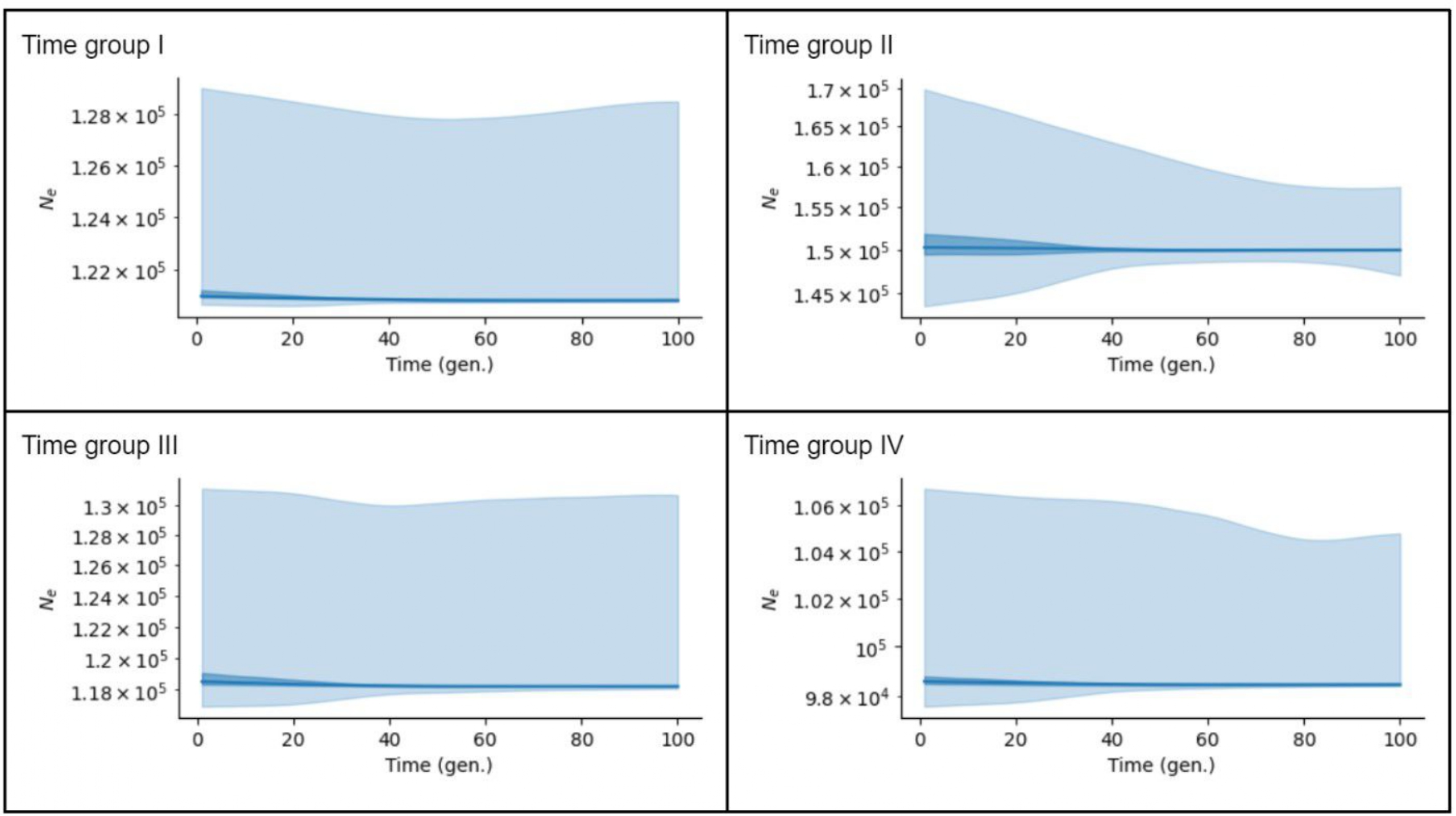


**Fig S7 Effective population size (Ne).** Effective population size (Ne) of the Sint-Truiden city center population inferred for each time group over its past 100 generations.


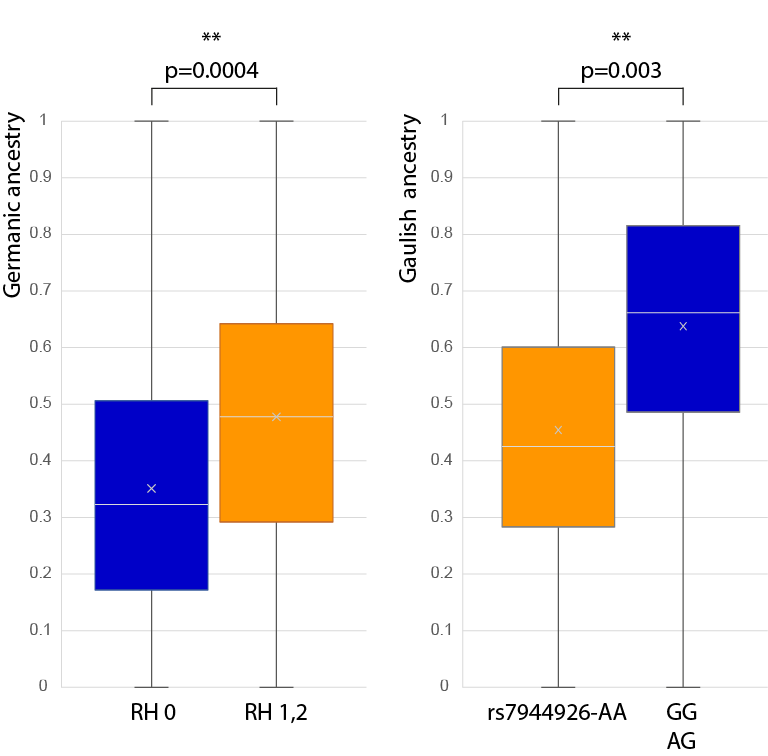


**Fig S8 Ancestry-related phenotype variation in Sint-Truiden burials.** RH 0 – individuals carrying no red hair causing alleles; RH 1,2 – individuals carrying at least one red hair causing allele in the *MC1R* gene among variants with strong effect - rs11547464_A, rs1805008_T, rs1805006_A, rs1805007_T, and rs1805009_C. rs7944926-AA – individuals homozygous for the A allele in the rs7944926 SNP in the *DHCR7* gene; rs7944926-AG and rs794492qleq6-GG – individuals carrying at least one G allele. Both red hair causing alleles and the rs7944926-G allele are associated with higher levels of vitamin D. **: p<0.01. Ancestry proportions are based on 2-way qpAdm analyses using Early Medieval Netherlands and Later Iron Age France as proxies for the Germanic and Gaulish ancestries, respectively. In both plots individuals predicted to have lower levels of vitamin D precursor 25(OH)D3 (calcidiol) in the blood are shown on the left and those with higher levels on the right side.


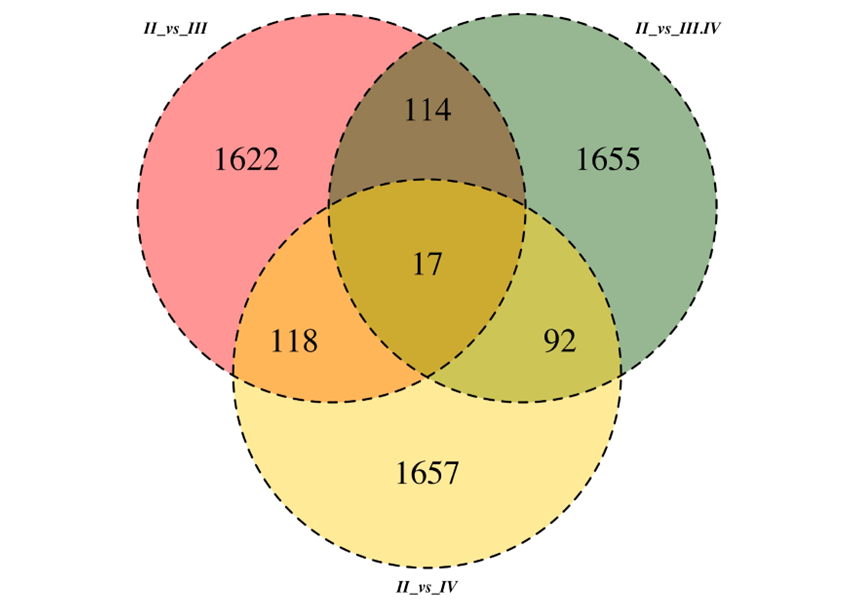


**Fig S9 Fst outlier analysis.** Venn Diagram of the variants with Fst over the 95th percentile of the genome-wide distribution in all comparisons between time groups.


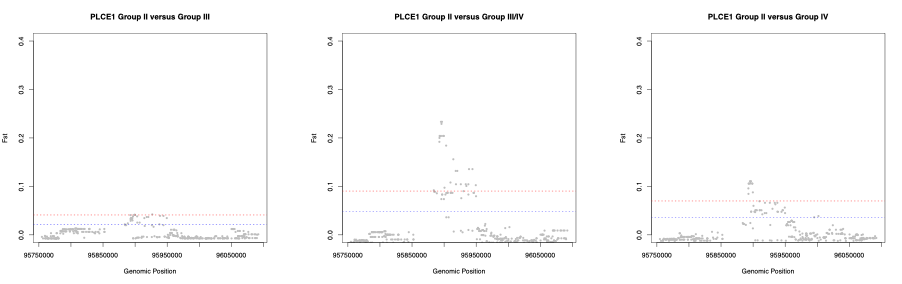


**Fig S10 *PLCE1* Fst values.** Single variant Fst for all variants present in *PLCE1* in all comparisons between time groups.


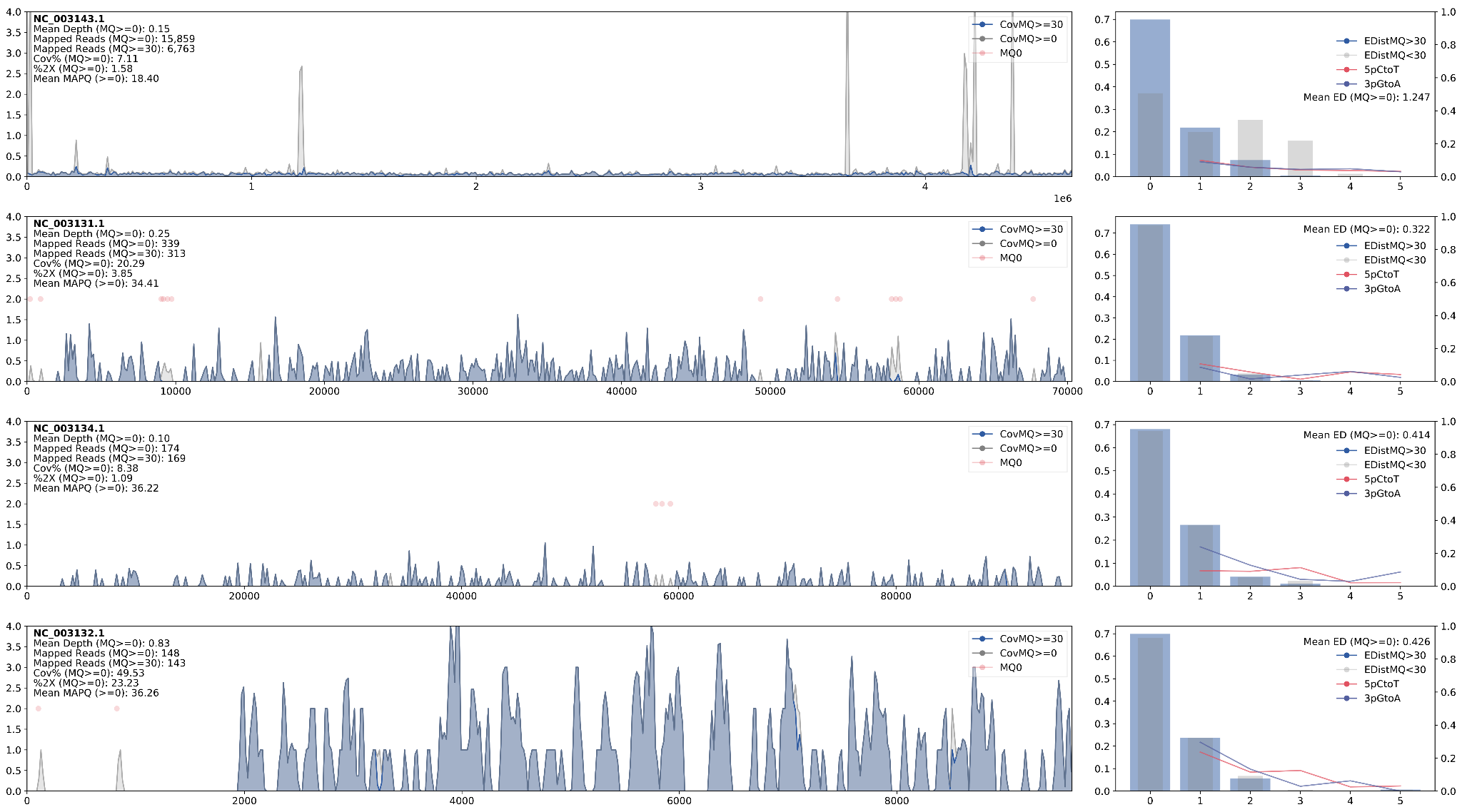


**Fig S11** Coverage plots for our mapping to the CO92 *Y. pestis* reference sequence. Intervals with reads under mapping quality of 30 are shown in light gray (depth of coverage shown on the y-axis and genome coordinates on the x-axis of the left plot). On the right, edit distances for the mappings are shown in a barplot (left axis) with light gray bars showing the percentage of reads under a mapping quality of 30 for each edit distance and blue bars showing the same for reads with mapping quality equal or above 30. The right axis shows the 5’ C>T (light red line; base 1 to 10) and 3’ G>A (light blue line; base -1 to -10) deamination frequency for each mapping.

**
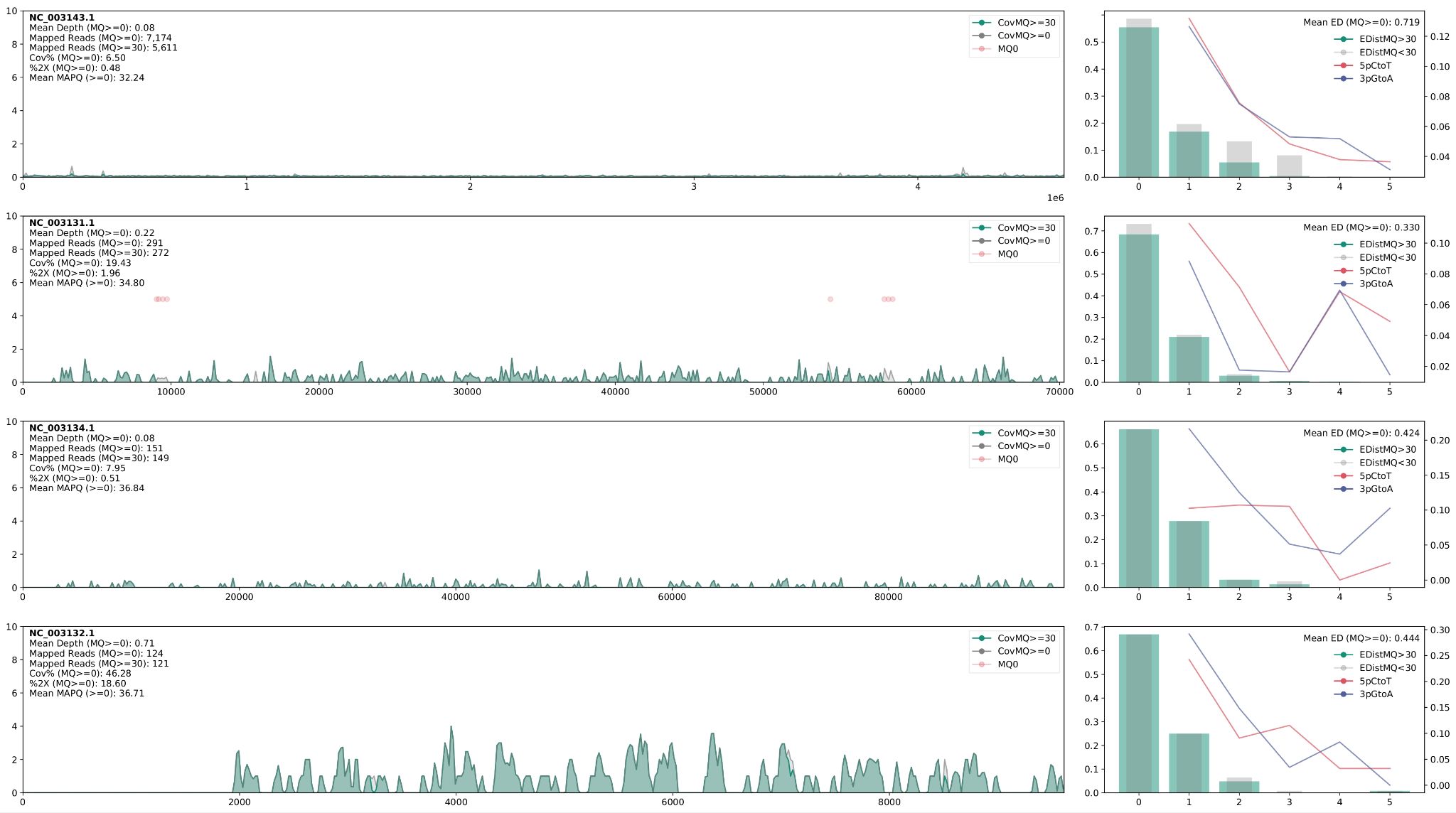
**

**Fig S12 Mapping plots of microbial genomes for sample ST1516.** Left: coverage plots, right: edit distance bar plots and deamination line plots.

References

[Bycroft C, Freeman C, Petkova D, Band G, Elliott LT, Sharp K, et al. The UK Biobank resource with deep phenotyping and genomic data. Nature. 2018 Oct 11;562(7726):203–9.](https://www.zotero.org/google-docs/?3x89ZR)

De Winter N. ARON rapport 1258 – Eindverslag Sint-Truiden Groenmarkt. Opgraving naar aanleiding van de herinrichting van de Groenmarkt, het Trudoplein, de Diesterstraat, de Plankstraat en de Meinstraat. 2023.

Fischer CE, Pemonge MH, Ducoussau I, Arzelier A, Rivollat M, Santos F, et al. Origin and mobility of Iron Age Gaulish groups in present-day France revealed through archaeogenomics. iScience. 2022 Apr;25(4):104094.

Garrison E, Marth G. Haplotype-based variant detection from short-read sequencing [Internet]. arXiv; 2012 [cited 2025 Apr 4]. Available from: https://arxiv.org/abs/1207.3907

Gretzinger J, Sayer D, Justeau P, Altena E, Pala M, Dulias K, et al. The Anglo-Saxon migration and the formation of the early English gene pool. Nature. 2022 Oct 6;610(7930):112–9.

Karmin M, Saag L, Vicente M, Sayres MAW, Järve M, Talas UG, et al. A recent bottleneck of Y chromosome diversity coincides with a global change in culture. Genome Res. 2015 Apr;25(4):459–66.

Keller M, Guellil M, Slavin P, Saag L, Irdt K, Niinemäe H, et al. A Refined Phylochronology of the Second Plague Pandemic in Western Eurasia [Internet]. 2023 [cited 2024 Sep 5].

Available from: <http://biorxiv.org/lookup/doi/10.1101/2023.07.18.549544>

Larmuseau MHD, Otten GPPL, Decorte R, Van Damme P, Moisse M. Defining Y-SNP variation among the Flemish population (Western Europe) by full genome sequencing. Forensic Sci Int Genet. 2017 Nov;31:e12–6.

Li H, Handsaker B, Wysoker A, Fennell T, Ruan J, Homer N, et al. The Sequence Alignment/Map format and SAMtools. Bioinformatics. 2009 Aug 15;25(16):2078–9.

Maréchal G. Lepra-onderzoek in Vlaanderen (XIVe -XVIe eeuw). Ann Société Belge D’ Hist Hôp Ann Van Belg Ver Voor Hosp. 1976;27–66.

McColl H, Kroonen G, Moreno-Mayar JV, Valeur Seersholm F, Scorrano G, Pinotti T, et al. Steppe Ancestry in western Eurasia and the spread of the Germanic Languages [Internet]. 2024 [cited 2024 Sep 9]. Available from: <http://biorxiv.org/lookup/doi/10.1101/2024.03.13.584607>

Mus O. De leprozerij, genaamd het Godshuis der Hoge Zieken te Ieper, 4 dln. Ieper; 1950.

Patterson N, Isakov M, Booth T, Büster L, Fischer CE, Olalde I, et al. Large-scale migration into Britain during the Middle to Late Bronze Age. Nature. 2022 Jan 27;601(7894):588–94.

Ringbauer H, Novembre J, Steinrücken M. Parental relatedness through time revealed by runs of homozygosity in ancient DNA. Nat Commun. 2021 Sep 14;12(1):5425.

Vanhoutte S, Bradt T, Vandenbulcke J. Beziect ende besmet metten lazers. Tijd-Schr. 2021;11(1):7–23.

Viaene A. Leprozen en leprozerijen in het oude Graafschap Vlaanderen. Tielt; 1962.

YFull | NextGen Sequence Interpretation [Internet]. [cited 2024 Sep 6]. Available from: <https://www.yfull.com/>
